# Oxidative Stress-Associated Apoptotic Responses Induced by *Lantana camara* L. Flower–Derived Zinc Oxide Nanoparticles in Human Non-Small Cell Lung Cancer (NCI-H460) Cells

**DOI:** 10.3390/molecules31162770

**Published:** 2026-08-09

**Authors:** Essa M. Sabi, Ahmed H. Mujamammi, Khalil I. Zarea, Ziyad M. Althafar, Khalid M. Sumaily

**Affiliations:** 1 Clinical Biochemistry Unit, Department of Pathology, College of Medicine, King Saud University, P.O. Box 2925, Riyadh 11461, Saudi Arabia; amujamammi@ksu.edu.sa (A.H.M.); ksumaily@ksu.edu.sa (K.M.S.); 2 Department of Physical Sciences, Chemistry Division, College of Science, Jazan University, P.O. Box 114, Jazan 45142, Saudi Arabia; kmudarmah@jazanu.edu.sa; 3 Department of Medical Laboratories Sciences, College of Applied Medical Sciences in Alquwayiyah, Shaqra University, Riyadh 11961, Saudi Arabia; zaldosari@su.edu.sa

**Keywords:** NCI-H460 cell line, lung cancer, zinc oxide nanoparticles, LC flower extract, oxidative stress, cell cycle arrest

## Abstract

Lung cancer remains a leading cause of cancer-related mortality worldwide, underscoring the need for safer and more effective therapeutic strategies. In this study, zinc oxide nanoparticles (ZnO NPs) were synthesized via a green, biogenic approach using *Lantana camara* L. flower aqueous extract and evaluated for their anticancer potential against human non-small cell lung cancer (NSCLC) NCI-H460 cells. The biosynthesized ZnO NPs were characterized using UV-visible spectroscopy, Fourier transform infrared spectroscopy (FTIR), scanning electron microscopy (SEM), Transmission electron microscopy (TEM), energy-dispersive X-ray analysis (EDX), X-ray diffraction (XRD) and particle size analysis, confirming the formation of nanocrystalline ZnO. LC-MS profiling of the *Lantana camara* flower extract revealed the presence of several bioactive phytochemicals, including phenolic compounds, terpenoids, fatty acids, and alkaloids, which may contribute to the reduction and stabilization of ZnO NPs during green synthesis. Cytotoxicity assessment of ZnO NPs using MTT and trypan blue exclusion assays revealed a dose-dependent reduction in cell viability, with an IC_50_ value of 50 µg/mL. Mechanistic investigations demonstrated that ZnO NP exposure induced significant oxidative stress, evidenced by increased nitric oxide, lipid peroxidation, and reactive oxygen species levels, along with depletion of intracellular glutathione. Apoptotic cell death was further confirmed by nuclear DNA fragmentation, mitochondrial membrane depolarization, and G0/G1 phase cell cycle arrest. Quantitative real-time PCR analysis revealed upregulation of the pro-apoptotic genes Bax and p53, accompanied by downregulation of the anti-apoptotic gene Bcl-2, indicating activation of a mitochondrial-dependent intrinsic apoptotic pathway. Collectively, these findings suggest that *Lantana camara* L. flower-mediated ZnO nanoparticles induced apoptotic responses associated with oxidative stress in NSCLC cells, highlighting their ability as an eco-friendly nanoplatform for further anticancer investigations.

## 1. Introduction

Lung cancer contributes substantially to global cancer-related mortality and continues to pose a serious public health burden. World Health Organization (WHO) reported (2022) that the recorded new cases were 2,480,675, with 1,817,469 deaths attributed to the disease [1,2]. The National Cancer Institute reports that the expected number of new cases in 2025 was nearly 226,650, accompanied by approximately 124,730 deaths [3]. The major causes include active and passive smoking, usage of pan and tobacco products, exposure to harmful and carcinogenic gases, air pollutants, etc. [4]. Several advanced treatments are available in lung cancer management, like surgical interventions, chemotherapy with cisplatin, paclitaxel, and etoposide, and radiotherapy techniques, including external beam and stereotactic body radiation therapy. Many molecular targeted therapies and immune checkpoint inhibitors are also available, such as tyrosine kinase inhibitors (TKIs), ALK inhibitors, EGFR inhibitors, and PD-1 inhibitors and PD-L1 inhibitors, respectively [1,5]. Despite these therapeutic advancements, lung cancer continues to account for nearly 11.1% of all newly diagnosed cancer cases, which contributes to about 20.2% of total cancer-related deaths worldwide, and the 5-year survival rate was 28.1% [1] after treatment, highlighting persistent limitations in conventional treatment strategies and underscoring the urgent need for more effective, safer, and innovative therapeutic interventions.

Metal oxide nanoparticles have captivated considerable focus in cancer research owing to their size, large surface area, electrical and optical properties, and structural stability, which collectively enhance their biomedical performance. Nanoparticles can easily penetrate the cellular membranes due to their small size and exhibit improved cellular internalization compared with conventional chemotherapeutic drugs [2]. Green-mediated fabrication of nanoparticles is increasingly preferred over chemically synthesized counterparts because of their eco-friendly nature, reduced toxicity, and improved biocompatibility. These surface-bound biomolecules can enhance nanoparticle stability, cellular uptake, and therapeutic efficacy, thereby improving anticancer potency [6,7]. Over the years, a variety of metal nanoparticles, including selenium, gold, zinc, titanium, silver, and copper, have been explored for cancer treatment and therapy owing to their unique physical–chemical and biological functions [8,9]. Among various nanomaterials, zinc oxide nanoparticles are considered relatively safe and have been broadly used in biomedical and consumer applications because of their biocompatibility and biodegradability [10]. ZnO nanoparticles are known to exhibit selective cytotoxicity toward cancer cells targeted through pathways that promote excessive reactive oxygen species (ROS) production, disrupt mitochondrial integrity and impair cellular energy metabolism, ultimately triggering programmed cell death [9]. These attributes make zinc-based nanoparticles promising candidates for the development of safer and more effective anticancer nanotherapeutics [8,11,12].

*Lantana camara* L. (LC) is a flowering shrub that belongs to the Verbenaceae family and is widely recognized as a weed. Native of the LC plant is South America and the Caribbean islands, but it is naturalized in more than 60 countries, which is commonly found in tropical, sub-tropical, and temperate regions, including Asia, Africa, Mexico, Florida, etc. The LC plant is distinguished by its various flower colors, such as orange, yellow and pink [13,14]. Leaves and seeds of LC contain lantadenes A and B, which are known to cause liver toxicity and cholestasis in animals upon ingestion. Although certain parts of *Lantana camara* exhibit toxic effects, the plant has long been utilized in traditional and folk medicine [15,16]. In traditional medicinal practices, LC has been used to manage diverse human ailments, including gastrointestinal disorders, malaria, swelling, wounds, skin inflammation, ulcers, respiratory infections, rheumatism, arthritis, hypertension, chickenpox, bilious fever and measles [17]. Scientific evidence reported that LC possesses a rich phytochemical profile and a wide range of pharmacological properties, such as antibacterial, antifungal, antifilarial activities, antiurolithiasis, antimutagenic, anticancer, and antimotility [18,19,20]. Notably, the flowers of LC are rich in bioactive compounds such as tannins, phenolics, and flavonoids, which are known for their antioxidant and anticancer potential [21].

This study utilized *Lantana camara* (LC) flower extract to fabricate ZnO nanoparticles (ZnO NPs) and investigated their anticancer potential on the human non-small cell lung cancer (NSCLC) cell line NCI-H460. Previous studies have primarily focused on the anticancer activity of *L. camara* leaves, seeds, oils, and flowers against lung cancer models such as the A549 cell line; however, there is very limited evidence regarding the anticancer properties of LC flower extract-mediated ZnO NPs. Based on the literature review, limited or no studies have reported the fabrication of ZnO NPs using LC flower extract for the evaluation of anticancer activity against NSCLC. Therefore, the novelty of the present work lies not merely in the use of a different plant part but in the integrated evaluation of the physicochemical characteristics and the underlying anticancer mechanisms, including oxidative stress, antioxidant defense, mitochondrial dysfunction, apoptosis, cell cycle arrest, and apoptosis-related protein expression.

## 2. Results

### 2.1. LC-MS Phytochemical Profiling of Lantana camara Flower Extract

**Figure 1:**
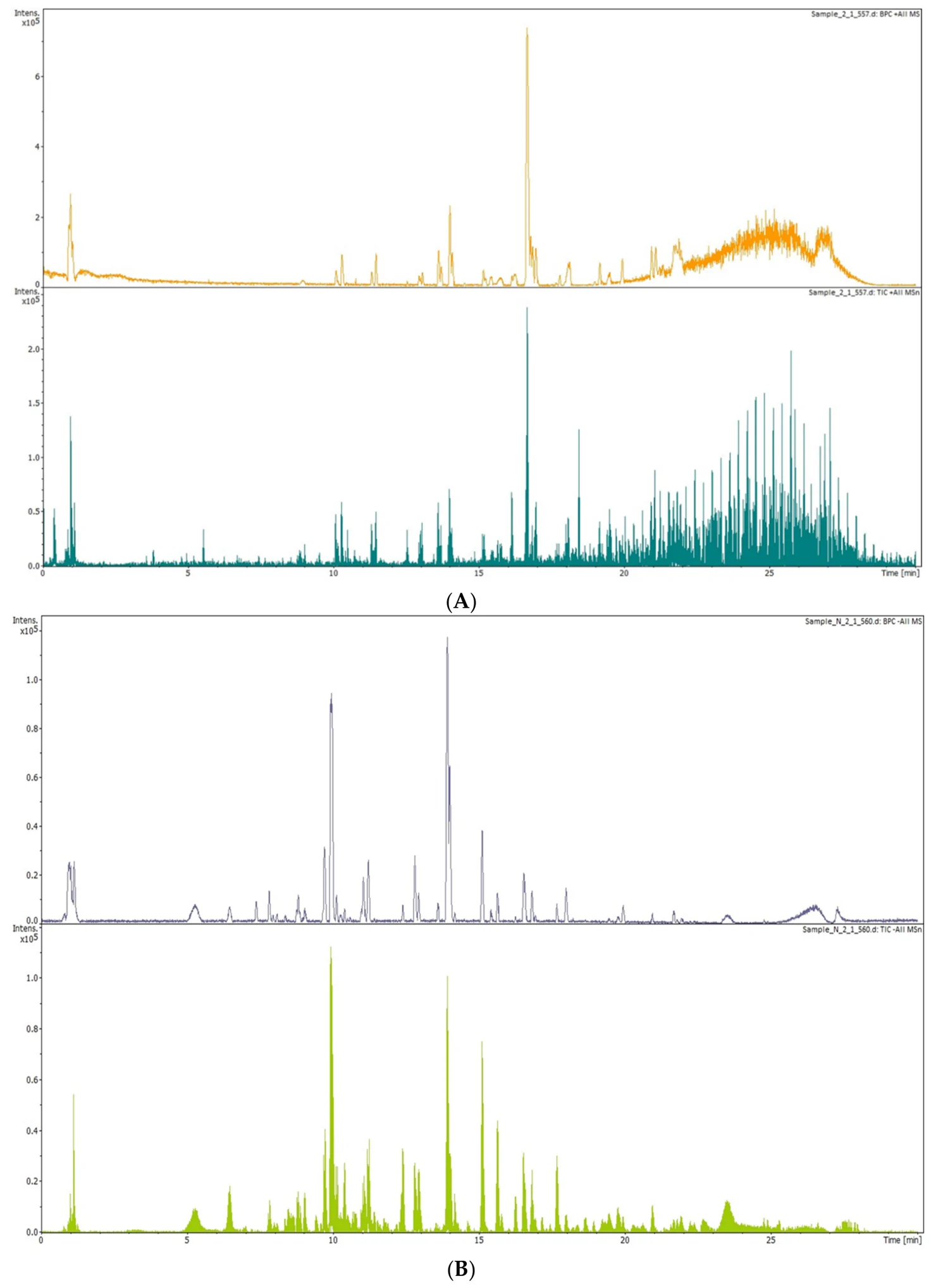
(**A**) Positive ionization mode of TIC and BPI chromatograms obtained from LC-MS for LCFAE. The peak denotes the presence of bioactive compounds detected with respect to the retention time. (**B**) Negative ionization mode of TIC and BPI chromatograms obtained from LC-MS for LCFAE. The peak denotes the presence of bioactive compounds detected with respect to the retention time.

LC-MS analysis was performed for the LCFAE to understand its phytochemical profile. The resulting chromatograms in positive and negative ionization modes are shown in Figure 1A,B, respectively. Furthermore, the details of the compound’s name, formula, chemical classes, etc., are summarized in Table 1 (positive) and Table 2 (negative), respectively. The analysis revealed a chemically diverse profile, with metabolites distributed across a wide retention time range, indicating compounds with varying polarities and structural complexities. In the positive ionization mode, several bioactive constituents were detected, including 6-gingerol, α-asarone, methyl jasmonate, trans, trans-farnesol, solasodine, oleamide, palmitic amide, dehydroabietic acid, tryptamine, and docosahexaenoyl glycine. Notably, long-chain fatty acid derivatives, such as tricosanoic acid methyl ester, showed relatively high signal intensity, suggesting their abundance in the extract. In the negative ionization mode, compounds such as (S)-2-hydroxyglutarate, 3-hydroxy-3-methylglutaric acid, 2,5-dihydroxybenzaldehyde, 9-cis-retinoic acid, iso-olomoucine, and pentalenic acid were identified, representing organic acids, phenolics and regulatory metabolites. Since authentic reference standards were not available, all identified phyto constituents are reported as tentatively identified.

### 2.2. Characterization

#### 2.2.1. UV Spectroscopic Analysis

**Figure 2:**
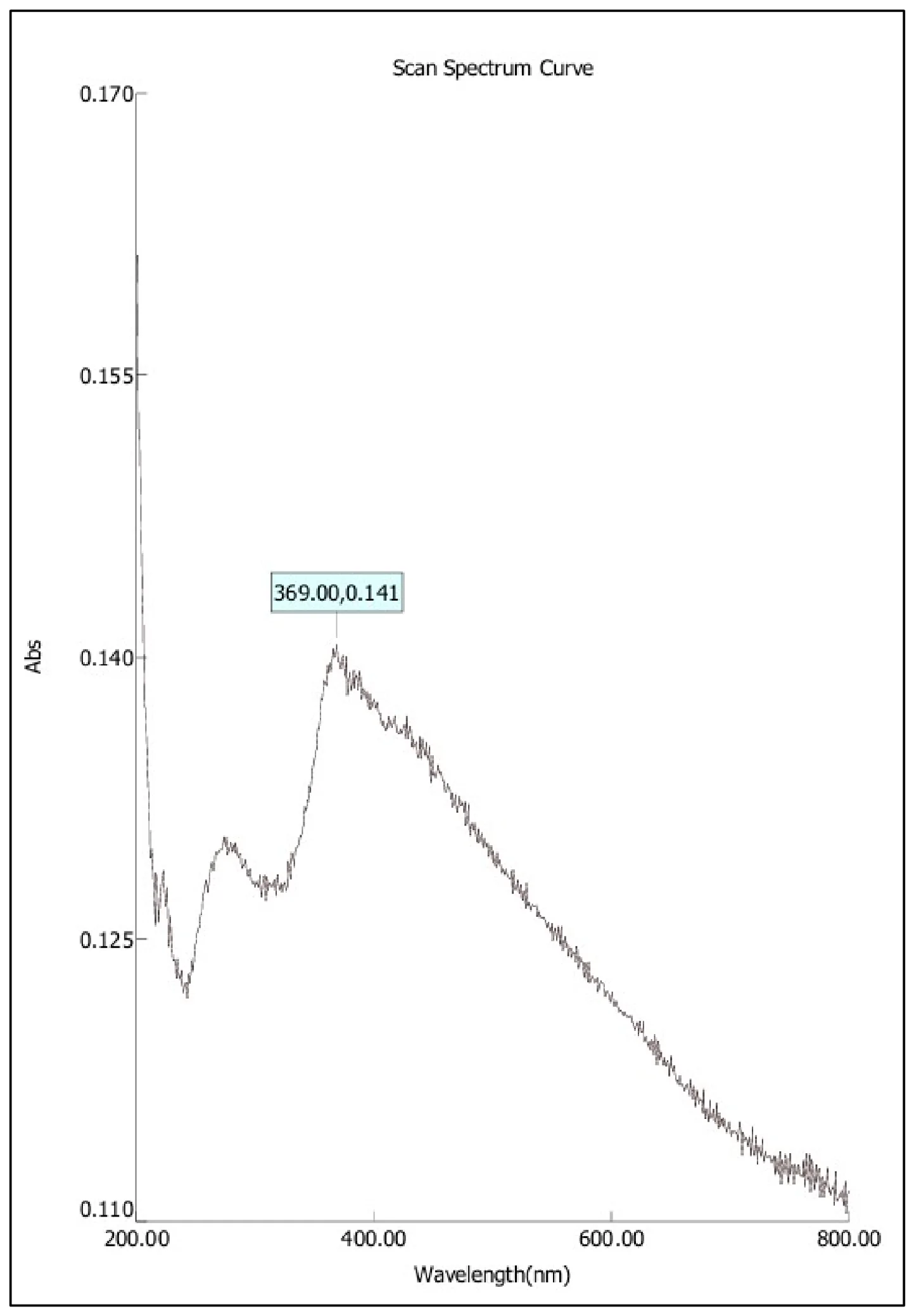
UV spectroscopic observation of LCFAE–ZnO NPs.

The UV-visible result of LCFAE-mediated ZnO NPs exhibited a prominent, sharp absorption peak at 365 nm (Figure 2). These absorption features indicate the formation of ZnO NPs.

#### 2.2.2. FT-IR Analysis

**Figure 3:**
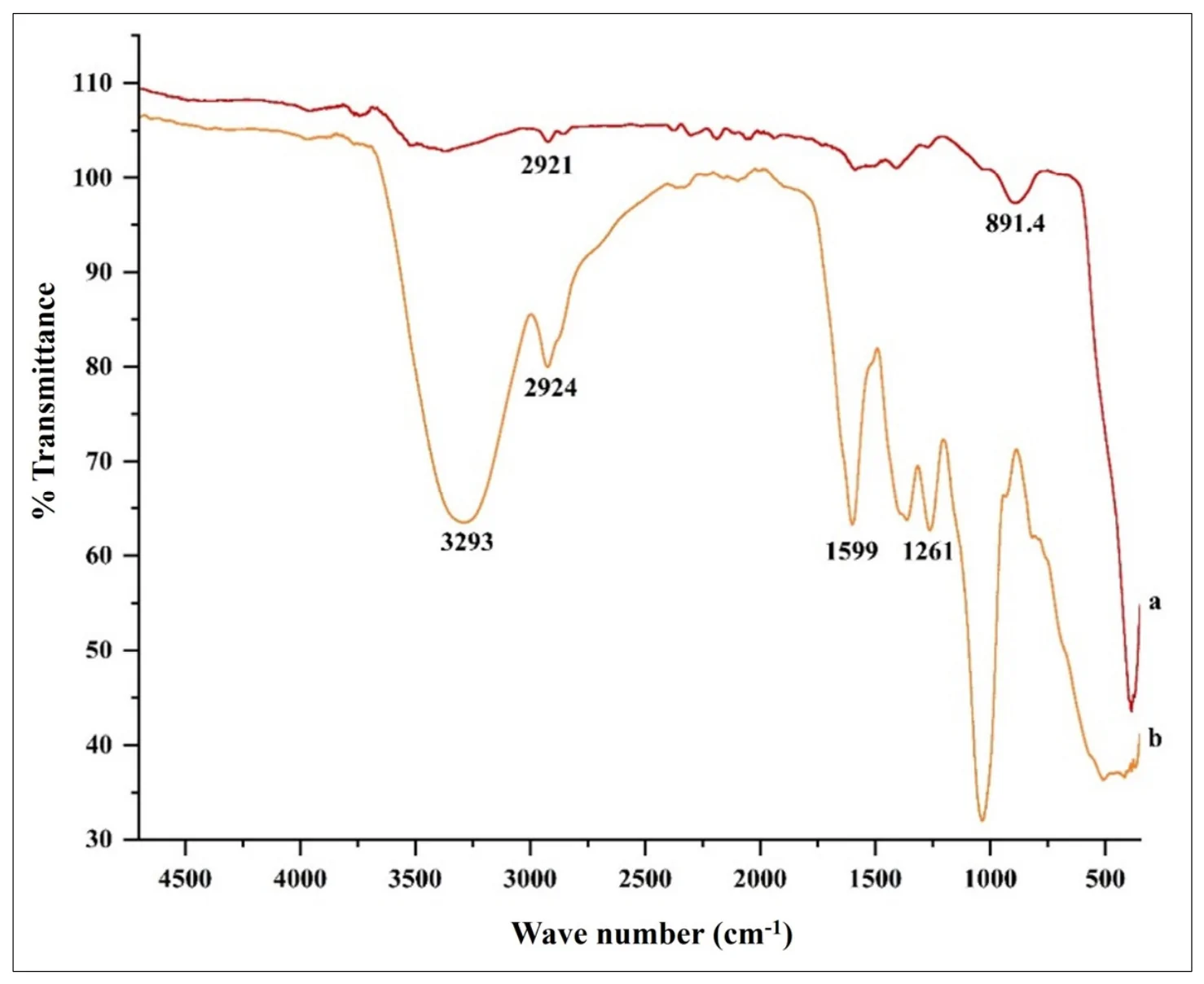
FT-IR analysis of LCFAE–ZnO NPs, denoted as (a), and *Lantana camara* plant aqueous extract, denoted as (b), showed 400–4500 cm^−1^ on the X-axis and % transmittance on the Y-axis.

FTIR spectra of plant-mediated ZnO NPs (a in Figure 3) and plant extract (b in Figure 3) were observed. The broad band at 3293 cm^−1^ represents phenolic and alcoholic compounds of O-H vibration. The band at 2921 cm^−1^ denotes the aliphatic group and represents C-H, which experienced a slight shift towards 2924 cm^−1^ at nanoparticle synthesis. The bands at 1599 cm^−1^ and 1261 cm^−1^ represent C=C and C-O bonds, indicating aromatic functional groups, respectively. The addition of peaks and absence of peaks denoted the successful synthesis of plant-mediated ZnO NPs. The 891.4 cm^−1^ peak denoted the synthesis of Zn-O stretching vibration.

#### 2.2.3. SEM and EDAX Analysis

**Figure 4:**
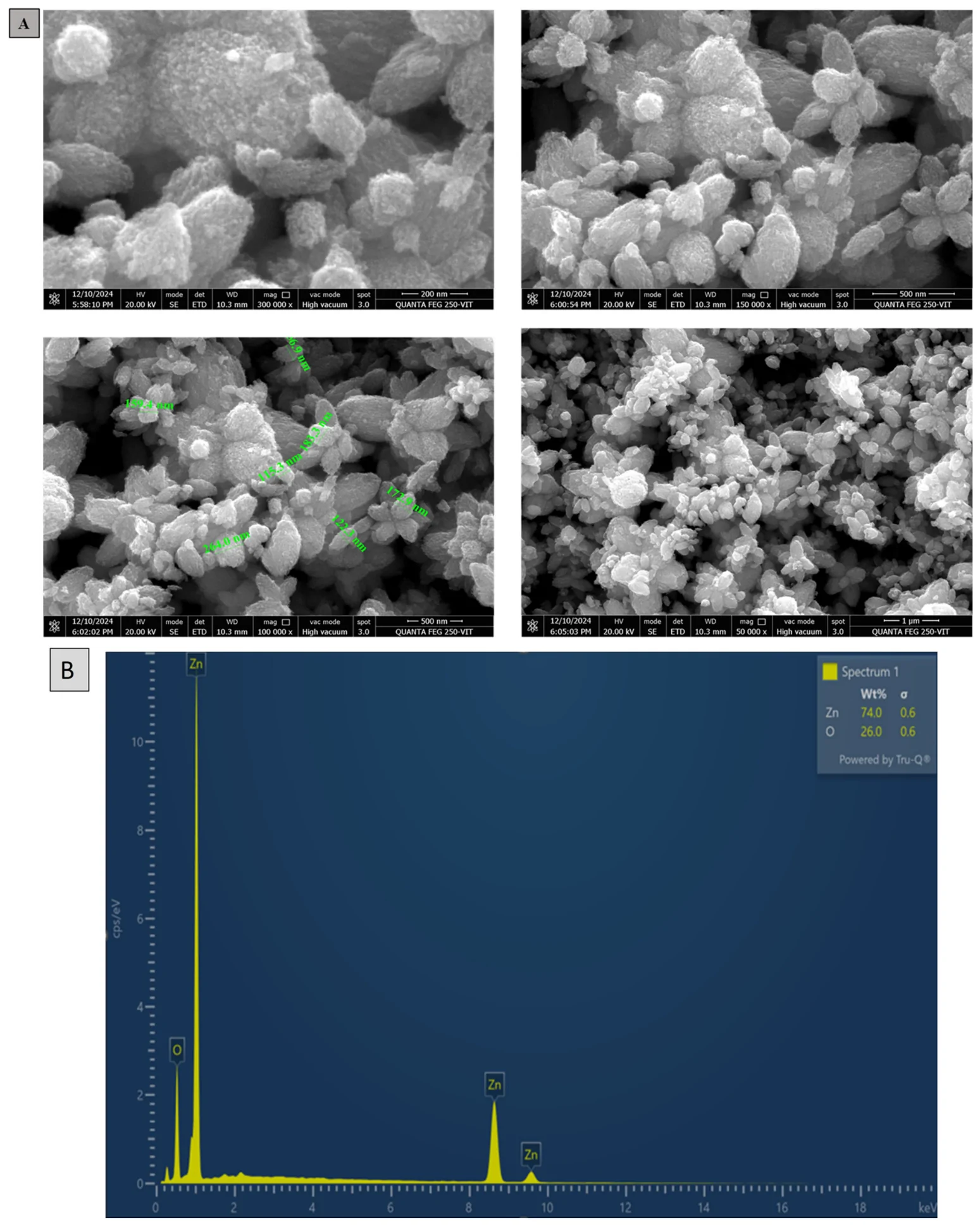
The surface of LCFAE–ZnO NPs was depicted in (**A**), and elemental composition was depicted in (**B**). SEM images were displayed at various scales, and nanoparticle sizes varied across different nanometer ranges.

Surface morphology was investigated by scanning electron microscopy, as demonstrated in Figure 4A. The green-synthesized ZnO nanoparticles revealed uneven polygonal shapes in the nanometer range. The formation of ZnO shows agglomerated and cluster-like structures. Some ZnO NPs exhibited flower petal-like structures. Elemental composition analysis (Figure 4B) revealed the major presence of Zn and O at 74.0% and 26.0%, respectively, clearly confirming the synthesis of ZnO NPs with high purity.

#### 2.2.4. X-Ray Diffraction (XRD)

**Figure 5:**
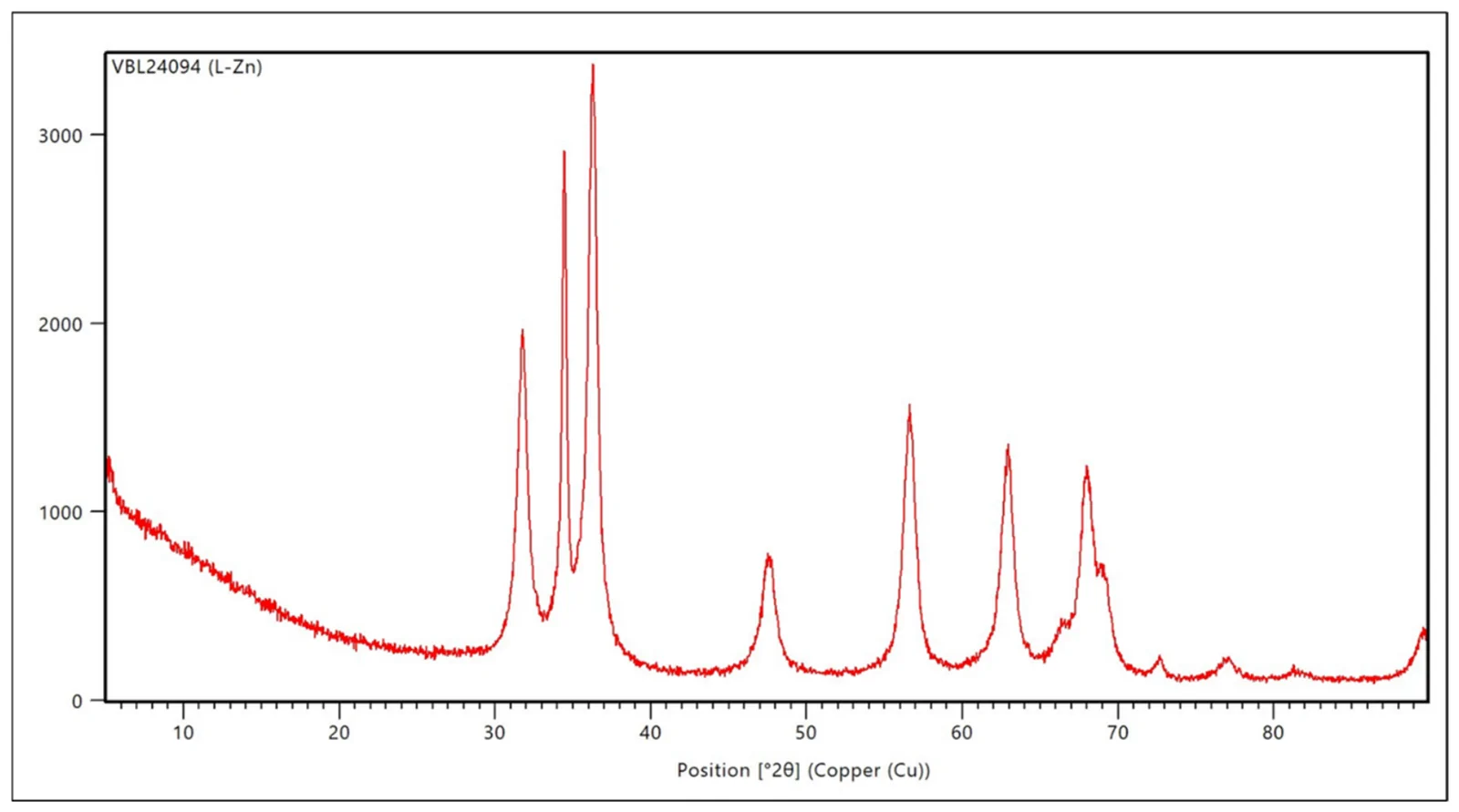
XRD: X-ray diffractometer of LCFAE–ZnO NPs was plotted between 2θ vs. counts. Different diffraction peaks responsible for ZnO NPs were observed in respective planes.

The crystallite nature of LCFAE-mediated ZnO NPs was studied and shown in Figure 5. The XRD graphical representation shows diffraction peaks at 31.7°, 34.4°, 36.2°, 47.5°, 56.6°, 62.8°, 66.3°, and 68.0° on the (100), (002), (101), (102), (110), (103), (200), and (112) planes, respectively. The peaks and planes reflect the ZnO Np’s Hexagonal wurtzite structure, and the peak broadening suggests the nanocrystalline nature. ZnO NPs synthesized in a highly purified form, confirmed by the absence of noisy peaks, which is in line with the EDAX analysis, showed only elements of Zn and O in high percentages.

#### 2.2.5. PSA and Zeta Potential

**Figure 6:**
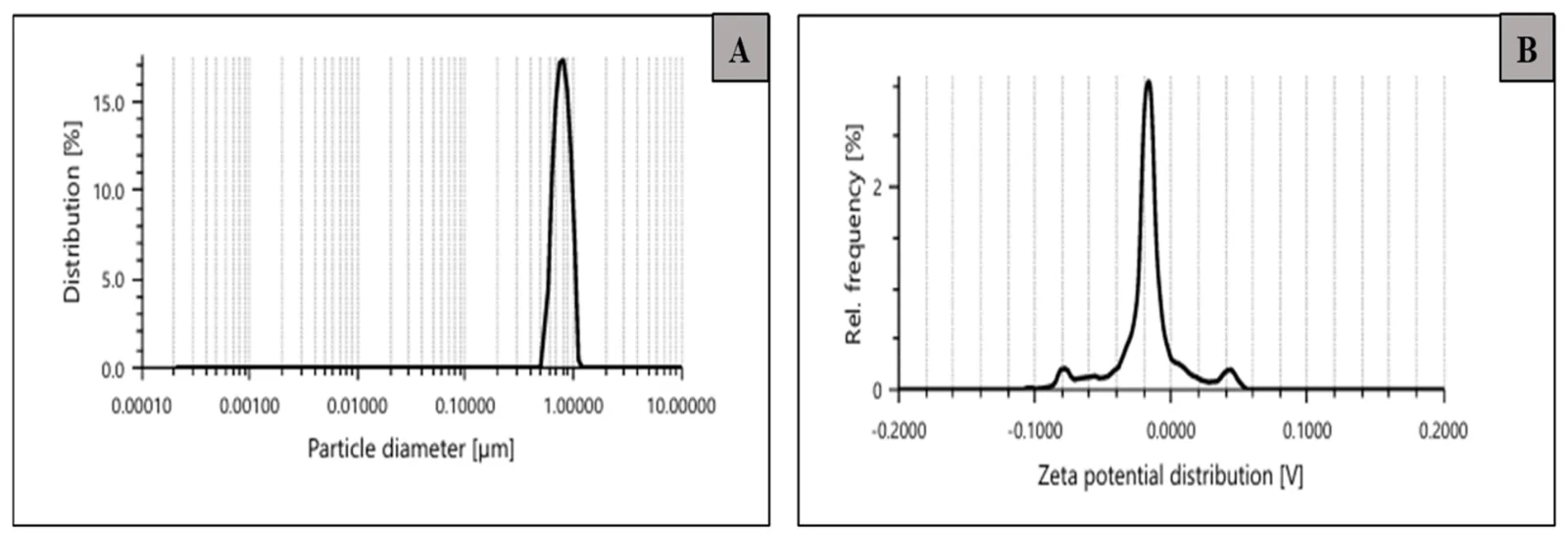
(**A**) denotes the particle size distribution of LCFAE–ZnO NPs and (**B**) denotes Zeta potential distribution of LCFAE–ZnO NPs.

Particle size and zeta potential of LCFAE-ZnO NPs were analyzed. Mean particle size was determined. The surface charge and colloidal nature were analyzed by a zeta-sizer. PSA and Zeta potential results are depicted in Figure 6A,B, respectively. Figure 6A shows the elevated single peak of hydrodynamic diameter was 1.0135 µm, centred at 0.7984 µm, indicating a narrow size distribution. This Mean particle size was observed due to the aggregation of particles in suspension. The polydispersity index (PDI) was 20.2%, indicating uniform particle distribution. The green synthesis of ZnO NPs reflects the large hydrodynamic size. Zeta potential analysis exhibited a mean zeta potential of LCFAE-mediated ZnO NPs of −0.0191 V, with a narrow distribution peak of −0.0166 V, which denotes a near-neutral zeta potential indicating moderate colloidal stability and negligible electrostatic stabilization.

#### 2.2.6. High Resolution—Transmission Electron Microscope

**Figure 7:**
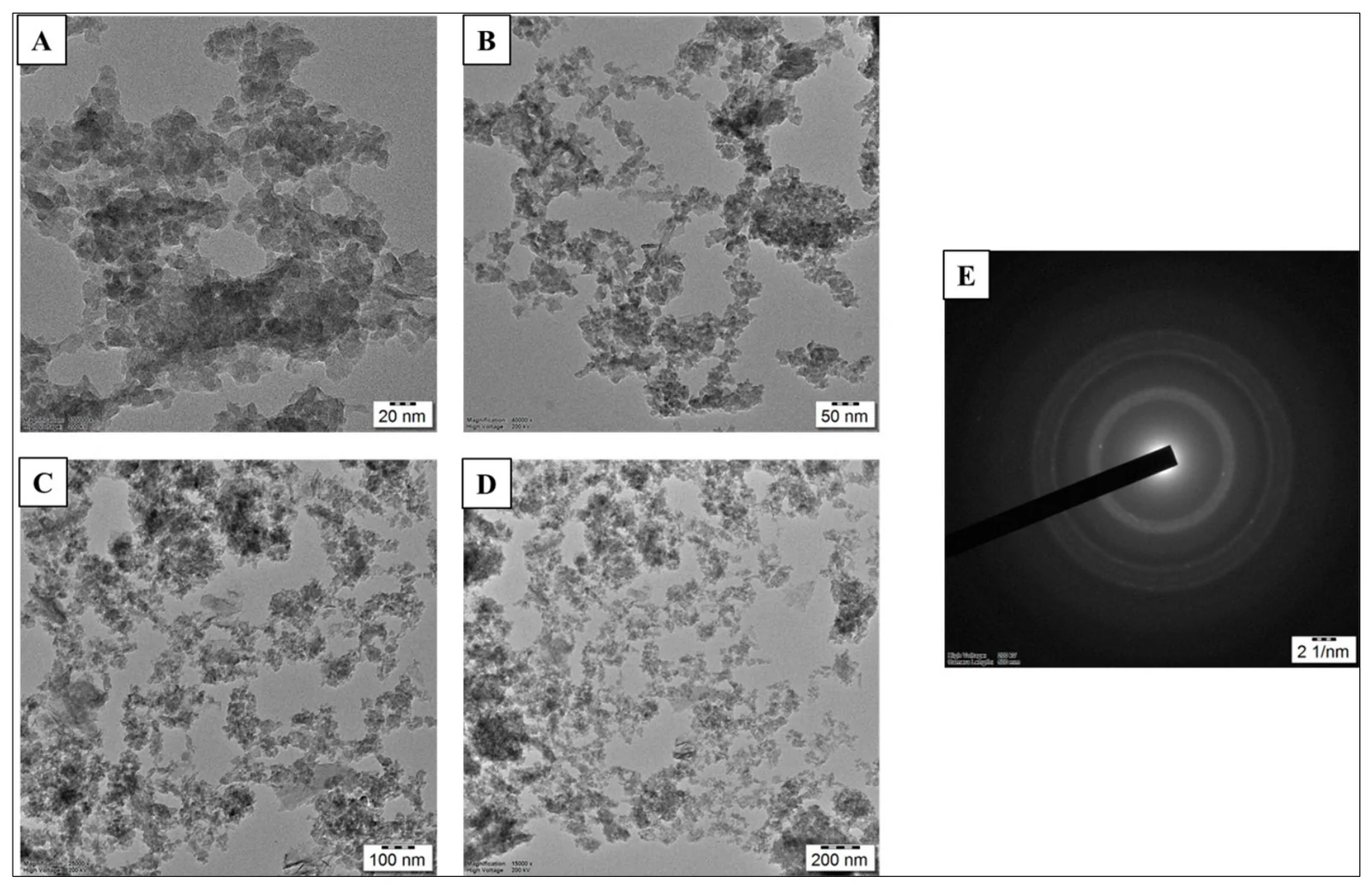
HR-TEM images. (**A**–**D**) show the primary nanoparticles of 20, 50, 100, and 200 nm, respectively. (**E**) shows the SAED pattern. The particles exhibited a spherical shape.

High-resolution transmission electron microscopy (HR-TEM) analysis was performed to further investigate the morphology and primary particle size of the biosynthesized ZnO nanoparticles. Figure 7 shows that the nanoparticles were predominantly spherical and had a size in the nanometer range. TEM micrographs revealed the agglomerated clusters. These interparticle interactions resulted from their high surface energy, a characteristic commonly observed in green-synthesized ZnO nanoparticles. The average primary particle size of TEM was calculated as 12.865 ± 4.457 nm (*n* = 60 particles). The SAED pattern exhibited a distinct diffraction ring, indicating the crystalline nature of the LCFAE-ZnO NPs.

### 2.3. Anti-Proliferative Property Assessment

#### 2.3.1. Cytotoxicity Assessment

**Figure 8:**
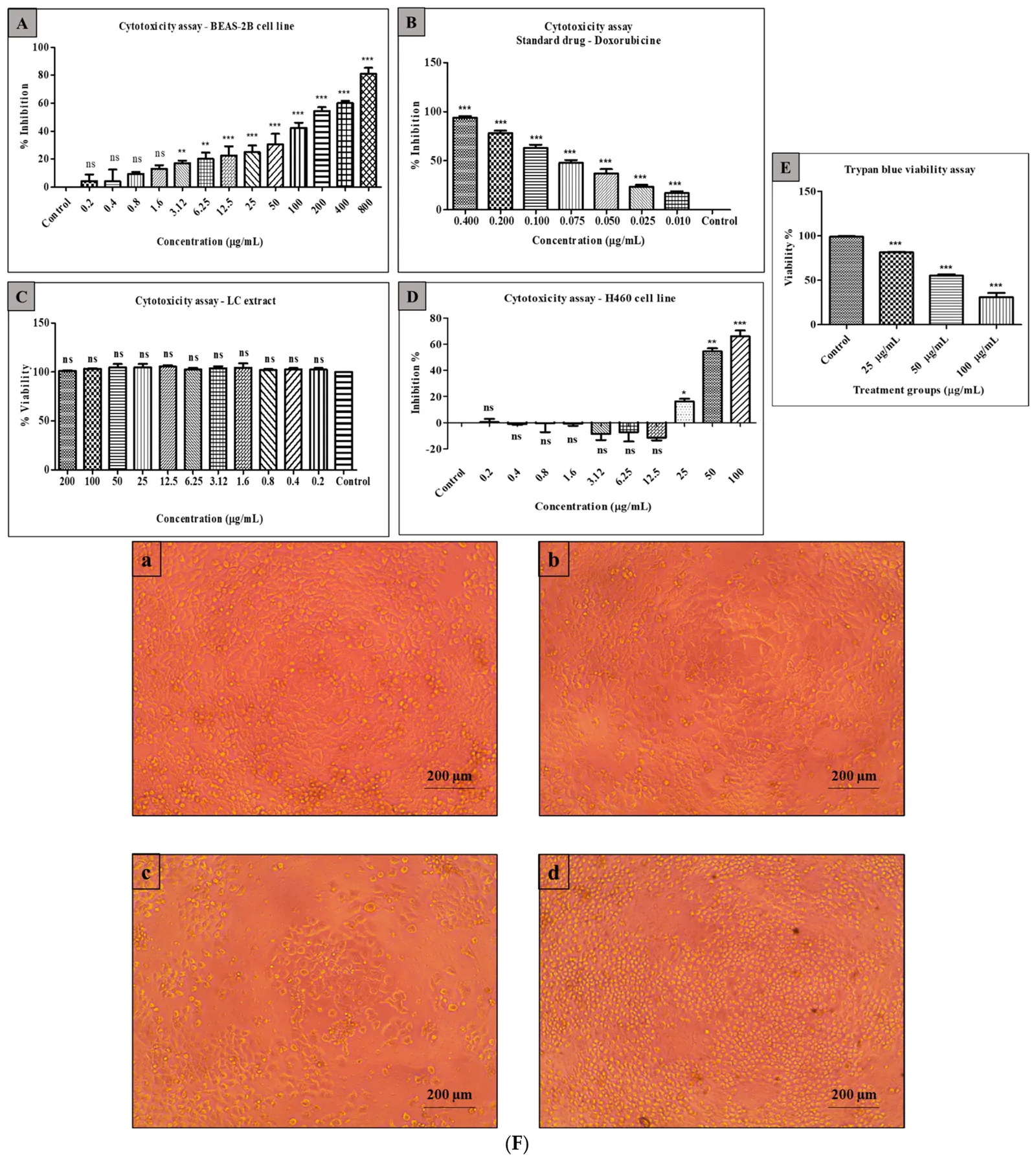
(**A**) represents the cytotoxicity values of LCFAE–ZnO NPs in the BEAS-2B cell line, (**B**) represents the cytotoxicity of the standard drug Doxorubicin against the H460 cell line, (**C**) represents the viability % of LC extract alone on H460 cells, (**D**) represents the LCFAE-ZnO NPs against the H460 cell line and (**E**) represents the trypan blue assay of LCFAE-ZnO NPs against the H460 cell line, denoted as % viability (ns—Non-significant, * *p* < 0.01, ** *p* < 0.001 and *** *p* < 0.0001). (**F**) Morphological observation: Morphometric assessment of LCFAE-ZnO NPs exposed H460 cell line is shown in the figure. Images (**a**–**d**) represent Control/untreated, 25 µg/mL, 50 µg/mL, and 100 µg/mL, respectively.

Cytotoxicity assessment of LCFAE–ZnO NPs was performed at concentrations ranging from 0.2 to 200 µg/mL in the BEAS-2B lung epithelial cell line. Figure 8A shows the significant cytotoxic effect of LCFAE–ZnO NPs on normal lung cells; there were significant dose-dependent changes in inhibition. Nearly 50% of the inhibition was observed at 200 µg/mL. Figure 8B shows the exposure of Doxorubicin, a standard drug against the H460 cell line, as a positive control in the range of 0.01–0.4 µg/mL. The graphical illustration exhibited nearly 95% inhibition at 0.4 µg/mL. Figure 8C shows the cytotoxicity of *Lantana camera* flower extract against the H460 cell line; no inhibition was observed up to 200 µg/mL. Figure 8D shows the LCFAE-ZnO NP exposure at 0.2–100 µg/mL against the NCI-H460 cell line; no inhibition was observed up to 12.5 µg/mL. However, cytotoxicity was observed at 25 µg/mL, with an inhibition of 16.18 ± 2.07%, which increased further at higher concentrations, reaching 54.43 ± 2.47% and 66.04 ± 4.41% at 50 and 100 µg/mL, respectively. The graph between concentration and the inhibition curve indicated that 50 µg/mL produced approximately 50% inhibition and was therefore considered the IC_50_ value. Hence, 25, 50, and 100 µg/mL were chosen for subsequent experiments. Lower concentrations showed no statistically significant difference compared to the untreated cells group (*p* > 0.05), whereas 25, 50, and 100 µg/mL demonstrated low, moderate, and high statistical significance, respectively, compared to the control, denoted as * *p* < 0.01, ** *p* < 0.001, and *** *p* < 0.0001. Ns—non-significant.

#### 2.3.2. Trypan Blue Exclusion (TBE) Assay

The TBE assay resulted in decreased cell viability in a dose-related manner upon treatment with LCFAE–ZnO nanoparticles (Figure 8E). The viability percentages at 25, 50, and 100 µg/mL were consistent with the cytotoxicity assay, showing a progressive reduction in viable cells with increasing concentrations. Lower concentrations showed no significant difference compared to the untreated group, while higher concentrations resulted in a substantial decline in cell viability.

#### 2.3.3. Morphological Assessment

The morphological disintegration in NCI-H460 cells upon treatment with LCFAE–ZnONPs at selected concentrations is shown in Figure 8F. The control cells (a) exhibited normal cellular morphology with intact cell structure and adherence. In contrast, cells treated with LCFAE–ZnO NPs displayed concentration-dependent morphological alterations. At 25 and 50 µg/mL (b and c), an increased number of dead cells and noticeable loss of normal cell morphology were observed. At 100 µg/mL (d), pronounced morphological changes, including cell rounding, cellular detachment, and membrane blebbing, were evident, indicating severe cytotoxic effects induced by LCFAE–ZnO NPs.

### 2.4. Antioxidant Enzyme Assessment

**Figure 9:**
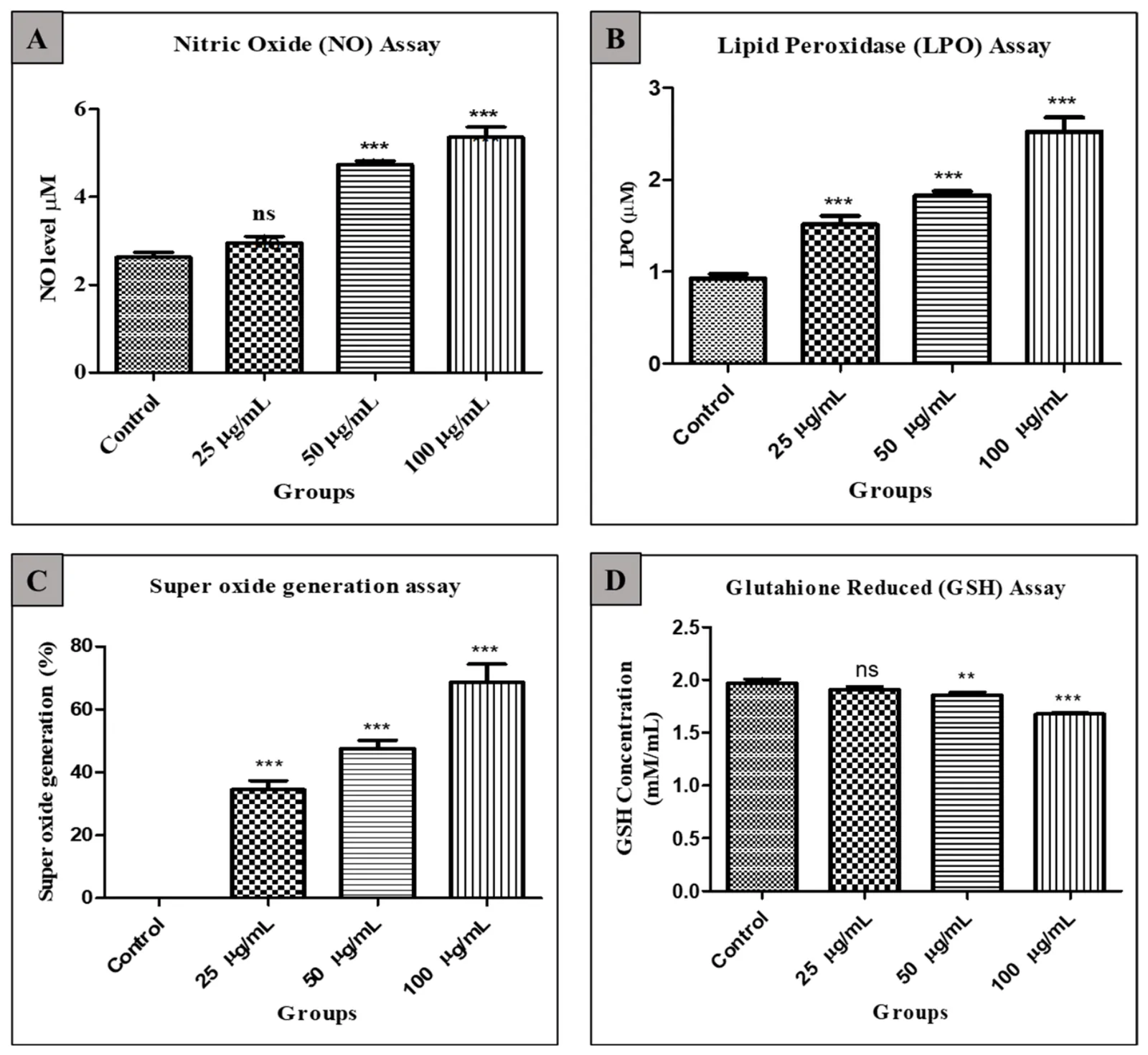
(**A**)—NO assay, (**B**)—LPO assay, (**C**)—superoxide generation assay, and (**D**)—GSH assay. In all the experiments, the treated group was compared with the untreated group by Tukey’s multiple comparison assay. Upregulated release of oxidative stress enzymes and downregulated release of antioxidant enzymes were observed in a dose-related manner. (ns—Non-significant, ** *p* < 0.01 and *** *p* < 0.001).

Nitric oxide (NO) release was measured in LCFAE–ZnO NPs exposed to various concentrations. Figure 9A shows a gradual, dose-related NO release. Control showed 2.625 ± 0.11 µM of NO release. In treated groups, NO release was observed as 3.375 ± 0.25, 5.888 ± 0.04, and 6.402 ± 0.20 µM for 25, 50, and 100 µg/mL of LCFAE–ZnO NPs. The 50 and 100 µg/mL groups showed highly significant differences from the untreated group (*** *p* < 0.001), whereas the 25 µg/mL group showed moderate significance (** *p* < 0.01). Similarly, LPO release increased significantly in a dose-dependent manner, whereas the control showed 0.929 ± 0.04 µM. Increased LPO release was observed at 25, 50, and 100 µg/mL of LCFAE–ZnO NPs, with values of 1.514 ± 0.09, 1.829 ± 0.04, and 2.524 ± 0.15 µM, respectively, as shown in Figure 9B. LPO release showed a highly significant difference between the treated and control groups (*** *p* < 0.001). The generation of reactive oxygen species was found to be consistent with the observed levels of nitric oxide (NO) and lipid peroxidation (LPO). Superoxide anion production increased in a concentration-dependent manner following treatment with LCFAE–ZnO nanoparticles. As per Figure 9C, cells treated with 25, 50, and 100 µg/mL of LCFAE–ZnO NPs exhibited superoxide anion generation levels of 34.48 ± 2.88%, 47.63 ± 2.55%, and 68.55 ± 5.84%, respectively, which were significantly higher compared to the untreated group (*** *p* < 0.001). These results indicated oxidative stress induction in NCI-H460 cells by LCFAE–ZnO nanoparticles in a dose-dependent manner.

In contrast, the non-enzymatic antioxidant, reduced glutathione (GSH), showed a decreasing trend in the treated groups in a dose-dependent manner. The GSH levels were recorded as 1.910 ± 0.03, 1.857 ± 0.02, and 1.675 ± 0.01 mM/mL for 25, 50, and 100 µg/mL of LCFAE–ZnO nanoparticles, respectively, whereas the untreated group exhibited a GSH level of 1.969 ± 0.03 mM/mL. As shown in Figure 9D, a dose-dependent reduction in GSH levels was observed. The 25 µg/mL group showed no significant difference compared to the untreated (ns, *p* > 0.05), while the 50 µg/mL group showed moderate significance (** *p* < 0.01), and the 100 µg/mL group exhibited high statistical significance (*** *p* < 0.001) compared to the control group.

### 2.5. Apoptosis Assessment

#### 2.5.1. Assessment of Nuclear DNA Damage and Mitochondrial Membrane Potential

**Figure 10:**
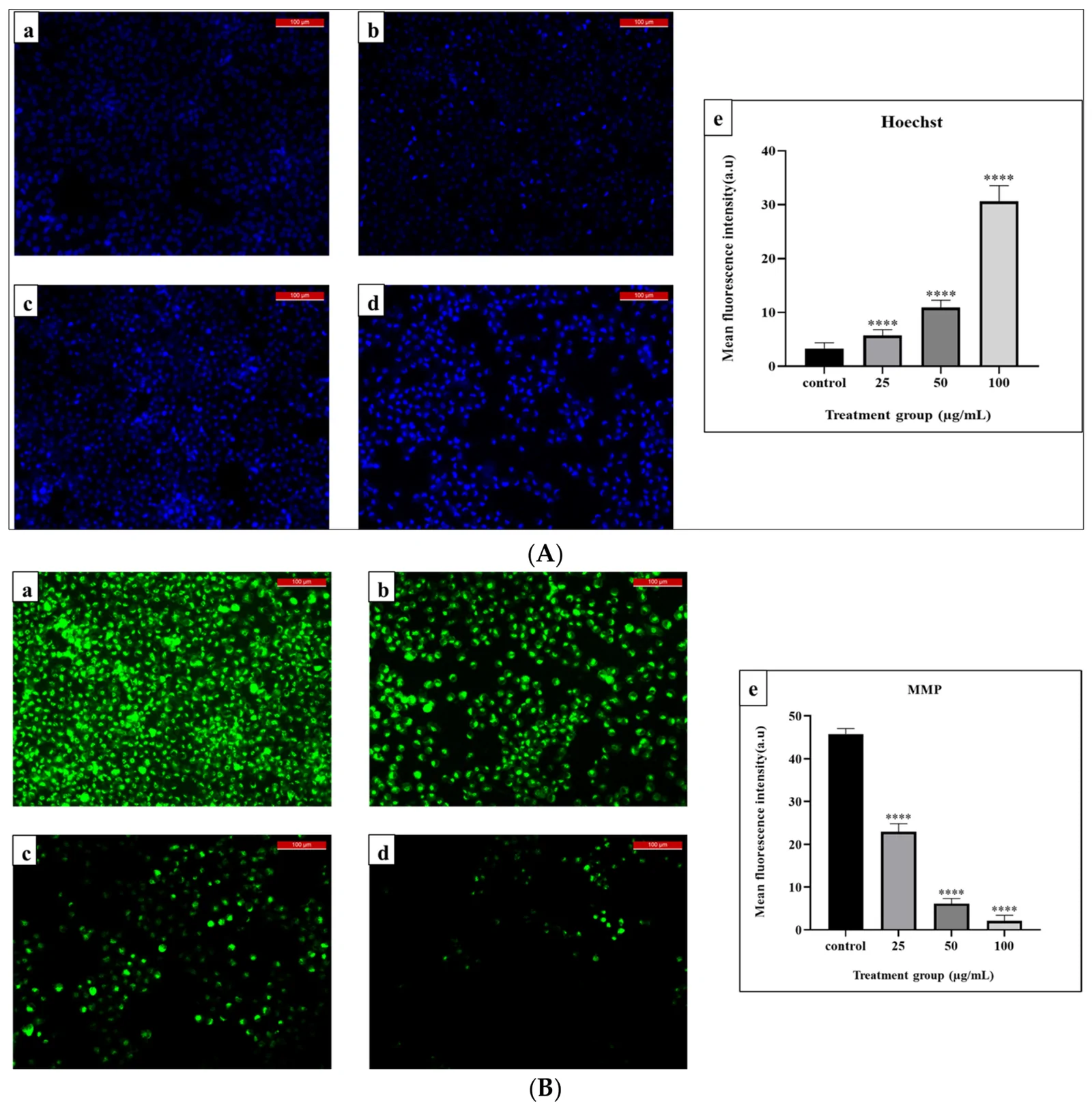
(**A**) H460 cell line treated and untreated groups exposed to Hoechst stain are shown in the figure. (**a**–**e**) represent Control/untreated, 25 µg/mL, 50 µg/mL, 100 µg/mL, and the densitometry graph of the Hoechst, respectively (*n* = 3). (**B**) H460 cell line treated and untreated groups exposed to Rhodamine123 stain are shown in the figure. (**a**–**e**) represent Control/untreated, 25 µg/mL, 50 µg/mL, 100 µg/mL, and the densitometry graph of the mitochondrial membrane potential (using Rhodamine123 stain), respectively (*n* = 3). (**** *p* < 0.0001). Scale bar: 100 µm.

Hoechst staining assay was performed to evaluate nuclear DNA integrity and chromatin stability in treated and untreated NCI-H460 cells. The fluorescence images obtained are shown in Figure 10A. The group of untreated cells (a) exhibited distinct cellular morphology with intact and uniformly stained nuclei. In contrast, cells treated with LCFAE–ZnO nanoparticles showed concentration-dependent nuclear alterations, including chromatin condensation and nuclear fragmentation. Treatment with 25, 50, and 100 µg/mL resulted in a progressive increase in dead cell count and fragmented nuclei with increasing concentration. Fluorescence intensity was measured using ImageJ software Version 1.54i and depicted in a graph (Figure 10A(e)).

Mitochondrial membrane potential (MMP) was investigated using Rhodamine staining in both treated and untreated cells (Figure 10B). The control group exhibited intense green fluorescence, indicating intact mitochondrial membrane integrity. In contrast, a dosage-dependent reduction in green fluorescence was noticed in LCFAE–ZnO NP-treated groups, indicating loss of mitochondrial membrane potential following exposure to different concentrations of LCFAE–ZnO nanoparticles. Fluorescence intensity was measured using ImageJ software Version 1.54i and depicted in a graph (Figure 10B(e)).

#### 2.5.2. Cell Cycle Analysis

**Figure 11:**
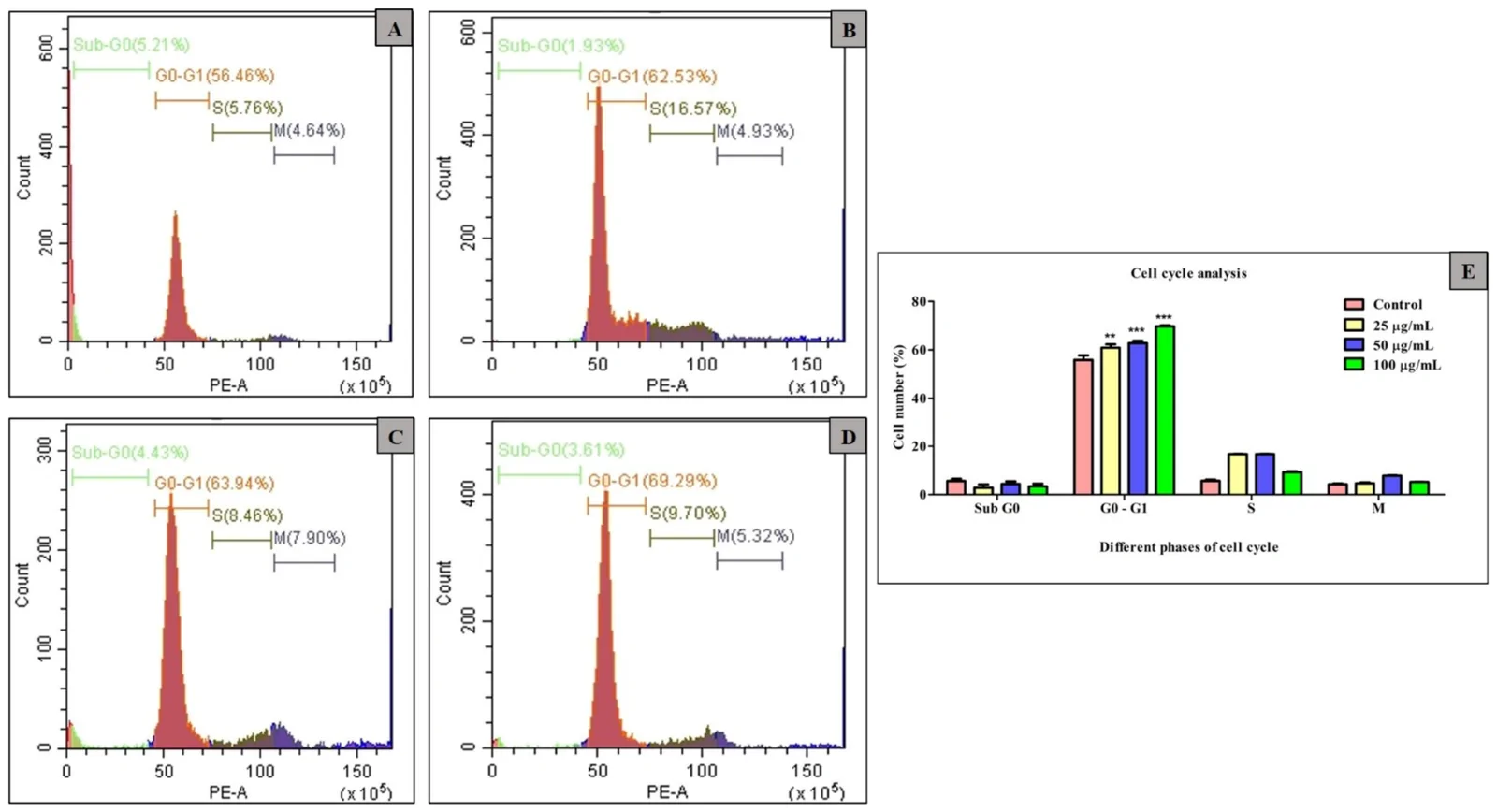
Cell cycle analysis by flow cytometry method: (**A**–**E**) represent Control/untreated, 25 µg/mL, 50 µg/mL, 100 µg/mL, and the quantitative graph, respectively. The data are provided as Mean ± SD in the graphical representation, *n* = 3. (** *p* < 0.01 and *** *p* < 0.001).

The effect of LCFAE–ZnO NPs on cell cycle progression was analyzed. Both untreated and treated NCI-H460 cells were subjected to flow cytometric analysis, and the distribution of cells across the cell cycle phases is shown in Figure 11. The treated groups exhibited a concentration-dependent increase in the percentage of cells in the G0/G1 phase. Notably, the highest concentration of 100 µg/mL resulted in 69.73 ± 0.417 of cells accumulating in the G0/G1 phase, indicating a pronounced cell cycle arrest at the G0/G1 checkpoint induced by LCFAE–ZnO nanoparticles.

#### 2.5.3. Gene Expression by qRT PCR

**Figure 12:**
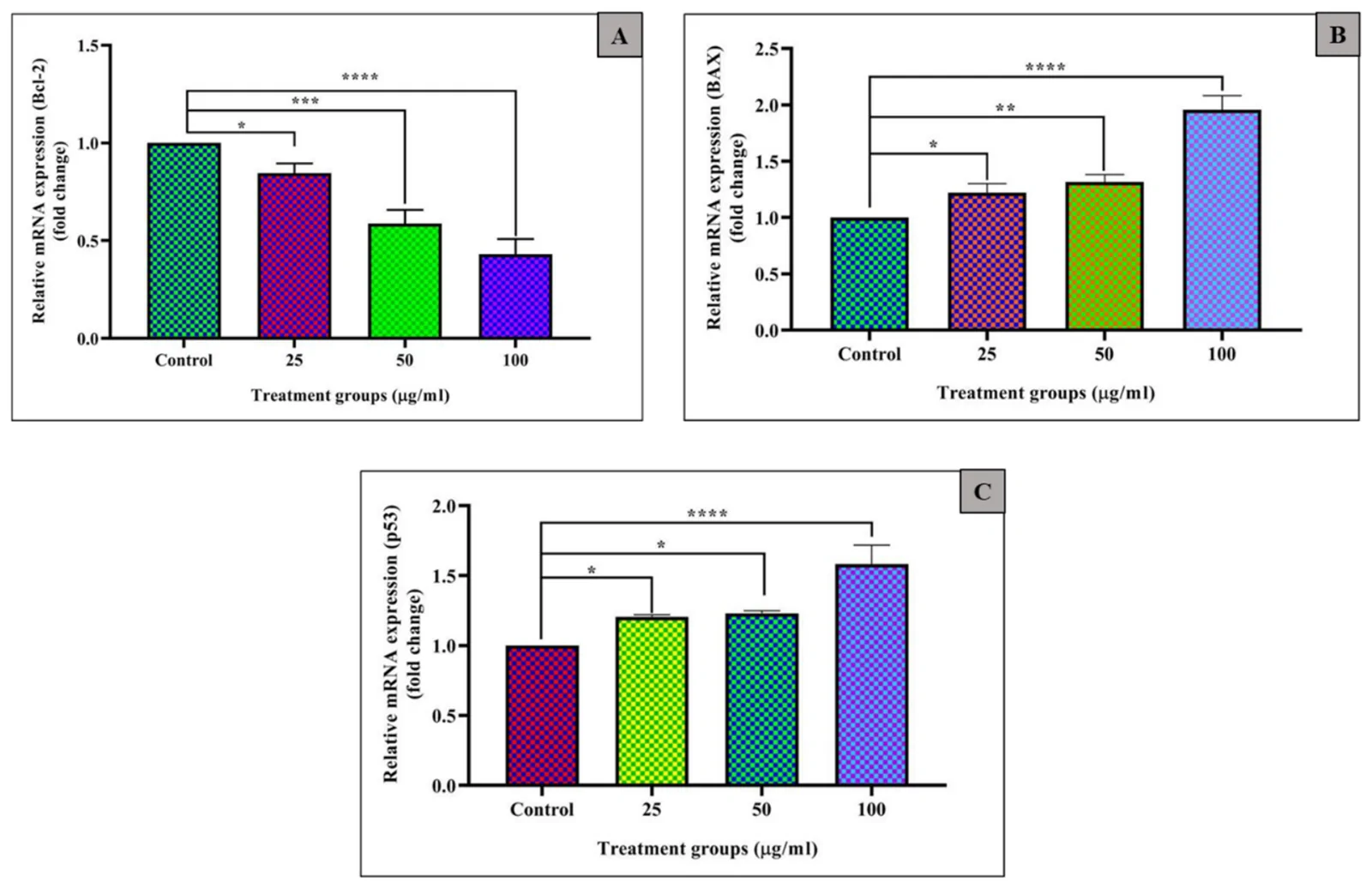
qPCR gene expression analysis: (**A**)—Bcl2, (**B**)—Bax and (**C**)—p53. Anti-apoptotic gene Bcl2 showed downregulated expression, and apoptotic genes Bax and p53 showed upregulated expression in a dose-related manner. (**** *p* < 0.0001, *** *p* < 0.001, ** *p* < 0.01, * *p* < 0.05).

The relative mRNA expression of Bcl-2, Bax, and p53 was analyzed to evaluate the effect of LCFAE–ZnO nanoparticles on apoptosis-related gene regulation (Figure 12A–C, respectively). The results revealed a dose-dependent modulation of these genes. Bcl-2, an anti-apoptotic gene, was significantly downregulated in the 50 and 100 µg/mL treated groups compared to the untreated, indicating suppression of cell survival signalling, whereas the 25 µg/mL group showed a comparatively lower and less significant reduction. In contrast, Bax, a pro-apoptotic gene, exhibited marked upregulation in the 100 µg/mL group and a moderately significant increase in the 50 µg/mL group, suggesting enhanced apoptotic activity at higher concentrations, while lower expression was noticed in the 25 µg/mL group. The tumour suppressor gene p53 was significantly upregulated in all treated groups, with the highest expression observed at 100 µg/mL. The 25 and 50 µg/mL groups also exhibited statistically significant, though comparatively lower, increases in p53 expression. One-way ANOVA and Tukey’s multiple comparison test disclosed significant differences between the control and treated groups (**** *p* < 0.0001, *** *p* < 0.001, ** *p* < 0.01, * *p* < 0.05; NS = non-significant).

#### 2.5.4. Western Blot Assay

**Figure 13:**
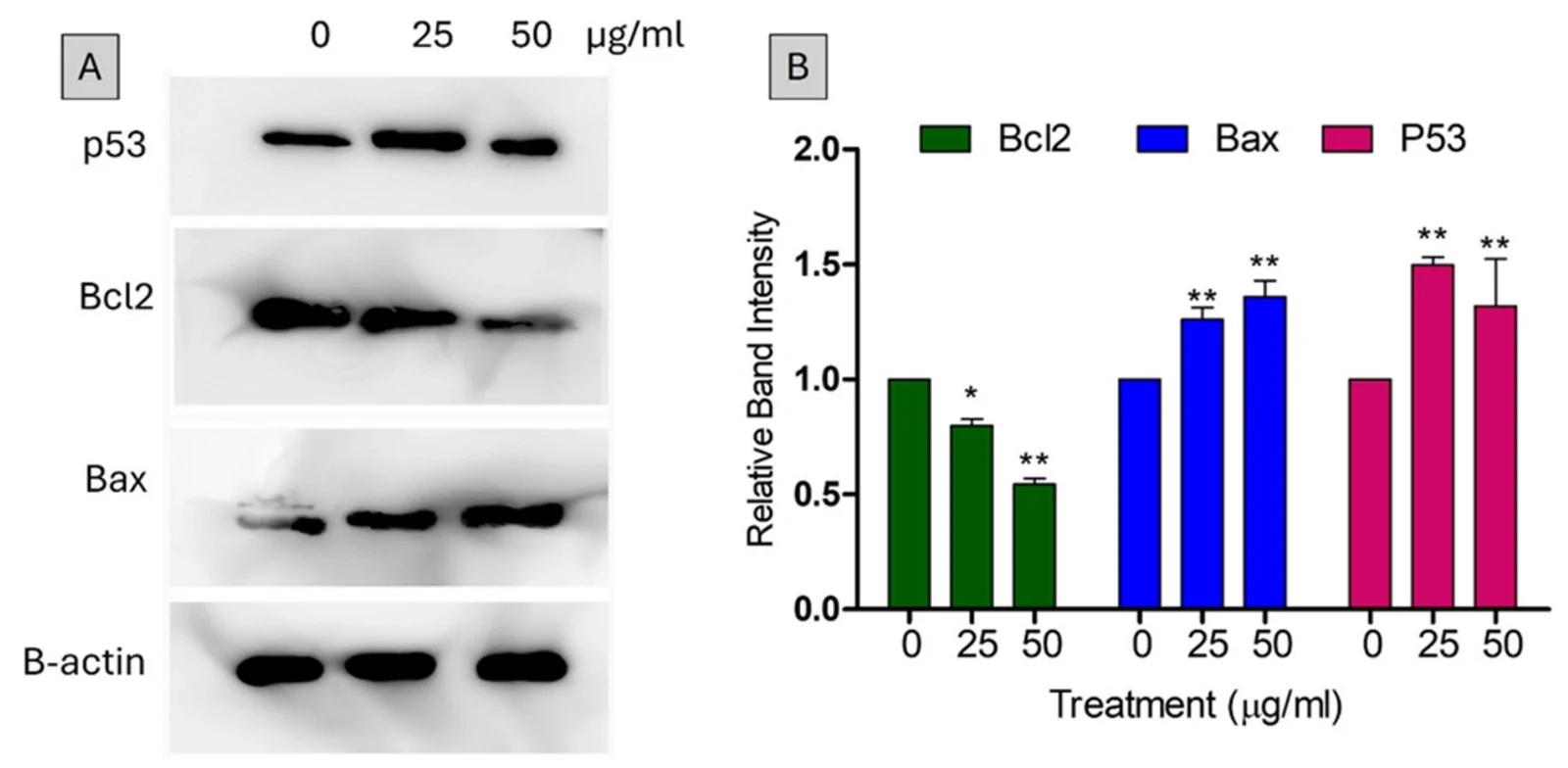
Effect of LCFAE-ZnO nanoparticles on apoptosis-related protein expression in H460 cells. (**A**) Western blot results showed expression of p53, Bcl2 and Bax proteins, while beta actin served as a housekeeping control. (**B**) Protein band intensity was measured, which showed that LCFAE-ZnO NPs significantly decreased the expression of Bcl2, while significantly upregulating Bax and P53. (** *p* < 0.01, * *p* < 0.05).

The effects of LCFAE-ZnO nanoparticles on apoptosis-related proteins were assessed by Western blotting. From Figure 13, it is clear that the LCFAE-ZnO nanoparticle has caused marked alterations in protein expression. A concentration-dependent reduction in protein expression was observed in the anti-apoptotic protein Bcl2. From the relative expression of 1 in the control group, 0.8- and 0.55-fold differences were observed at 25 and 50 µg/mL, respectively. In contrast, the pro-apoptotic protein Bax was significantly increased in both treated conditions, i.e., its relative expression increased to 1.25- and 1.38-fold at 25 and 50 µg/mL, respectively. Meanwhile, p53 protein expression was significantly upregulated to 1.5-fold at 25 µg/mL and 1.3-fold at 50 µg/mL compared to the control group.

## 3. Discussion

Lung cancer remains a major global health burden, as the number of new cases is increasing day by day due to delayed diagnosis and poor therapeutic outcomes. In most cases, the disease is detected at advanced stages, leading to metastasis, which results in limited and unsuccessful treatment, which has a great impact on patient survival [22]. Although multiple advanced therapeutic strategies are currently available, their effectiveness is often compromised by systemic toxicity, treatment resistance, disease recurrence, and the need for prolonged monitoring. These limitations highlight the intense requirement for alternative approaches that are not only potent but also safer and more selective. In this context, *Lantana camara* flower-mediated ZnO nanoparticles have attracted considerable research interest owing to their biocompatibility, reduced adverse effects, and ability to modulate cancer-associated cellular pathways, offering improved lung cancer management [23,24]. Green-synthesized ZnO Nps prepared using different plant extracts have previously been reported to exhibit cytotoxic activity against various lung cancer cell lines through oxidative stress-associated apoptotic mechanisms. The present study demonstrated comparable biological responses in NCI-H460 cells. The significance of the present work lies not in proposing a previously unknown ZnO-mediated mechanism but in providing an initial in vitro research of LC flower extract-mediated ZnO Nps in the NCI-H460 cells. These research findings will support and provide evidence for future in vivo translational investigations.

*Lantana camara* (LC) is a well-known plant with a long history of use for its pharmacological properties. All the parts of the LC plant, including leaves, seeds, fruits, flowers and essential oil, are known to possess a rich phytochemical profile and numerous studies have reported the cytotoxic potential of each part of the LC plant against lung cancer models such as the A549 cell line [15]. LC leaf and fruit-mediated synthesis of various metallic nanoparticles has been extensively explored, with several reports demonstrating their cytotoxic efficacy against lung cancer cells [6,25]. In contrast, flower-mediated nanoparticle synthesis using LC remains comparatively underexplored. Flowers of LC possess higher profile phytochemicals, including tannins, polyphenols, cardiac glycosides, flavonoids, anthocyanins, alkaloids, volatile oils, and some secondary metabolites [13,15,16,21]. Limited studies have reported on copper oxide NPs fabricated using LC flower extract and evaluated their cytotoxic effects against breast cancer [26]. Nickel oxide NPs derived from LC flowers have primarily been investigated for antibacterial and photocatalytic applications [27]. A few reports have described the zinc oxide NPs synthesis using LC flower extract, focusing on antibacterial potency, wound-healing potential, photocatalytic performance, and anticancer effects against the A549 lung cancer cell line. Therefore, the novelty of the present work lies not merely in the use of a different plant part but in the integrated evaluation of the physicochemical characteristics and the underlying anticancer mechanisms, including oxidative stress, antioxidant defense, mitochondrial dysfunction, apoptosis, cell cycle arrest, and apoptosis-related protein expression.

LC-MS analysis of the Lantana camara flower extract revealed the presence of a wide range of phytochemical constituents. They include many classes of compounds such as phenolic compounds, phenylpropanoids, terpenoids, fatty acids, and alkaloids. Phenolic derivatives present in the extract are well known for their antioxidant activity and their ability to donate electrons, which facilitates the reduction of metal ions during plant-mediated nanoparticle synthesis and contributes to nanoparticle stabilization [28]. In addition, terpenoid constituents may act as reducing agents, helping to regulate nanoparticle formation and prevent aggregation [29]. Fatty acids and related lipid derivatives further enhance stability through hydrophobic interactions that support structural integrity, while the presence of alkaloids and other nitrogen-containing compounds reflects the chemical diversity of the extract and may also contribute to stabilization and biological activity [30]. Overall, these results suggest that the plant extract has a mixture of bioactive chemicals, which can act as reducing and stabilizing agents during the green synthesis of LCFAE–ZnO NPs. The current investigation was carried out to provide preliminary evidence of the leaf extract in anticancer activity against H460 cells. The lack of quantitative profiling and standardization of major bioactive constituents needs to be carried out in future studies for translating the compounds to clinical application. Residual plant species may be the reason behind the observed cytotoxicity and antioxidant properties in the present study, but the exact surface chemistry of the nanoparticle has to be included in future investigations to understand the presence of phytochemicals.

Zinc oxide nanoparticles are generally reported as safe (GRAS) by the FDA, and they also have desirable physical, electrical, optical, morphological, and surface properties suitable for biological applications. Zinc oxide nanoparticles were synthesized using the LC flower aqueous extract, which facilitates the presence of phenols, flavonoids, tannins, and quinones in higher quantities than the solvent extract [18]. Characterization results disclosed the successful synthesis of ZnO NPs, with UV-visible spectroscopic analysis showing a sharp absorption peak at 365 nm, indicating the signature ZnO peak. A similar peak was reported in *Solanum procumbens*-mediated ZnO Nps synthesis. Birhan and Misskire reported that the λmax value of green-synthesized ZnO Nps peaks ranges between 280 and 380 nm [31,32]. FTIR spectral analysis revealed the involvement of phytochemicals such as hydroxyl 3293 cm^−1^, aliphatic 2921 cm^−1^, and aromatic moieties 1599 and 1261 cm^−1^, with notable shifts in the plant extract that are involved during the nanoparticle synthesis. However, because the nanoparticles were calcined at 400 °C for 2 h, the present study does not claim that these organic constituents remain on the surface of the final ZnO Nps. Additional surface-sensitive characterization techniques will be involved in future investigations that would be required to confirm the presence of residual organic species after calcination. The presence of Zn–O stretching vibrations in the lower wavenumber region, such as 891.4 cm^−1^, confirmed ZnO nanoparticle formation. A similar FT-IR pattern was reported for ZnO NPs synthesized from *Euphorbia retusa* [33]. Scanning electron microscopy revealed agglomerated, irregularly spherical nanoparticles, and EDX confirmed the pure synthesis of ZnO NPs [30]. X-ray diffraction analysis revealed distinct diffraction peaks characteristic of the hexagonal wurtzite structure of ZnO, confirming the crystalline nature and phase purity of the nanoparticles. The absence of impurity peaks indicated successful synthesis. A similar XRD pattern with a single-peak purity and the characteristic ZnO NP planner was reported for Lantana camara leaf-mediated ZnO NP synthesis [34]. Particle size analysis showed a larger hydrodynamic diameter, which can be attributed to the presence of hydration layers and may be due to the high surface energy. ZnO nanoparticles synthesized using *Annona squamosa* leaf extract and *Garcinia mangostana* leaf extract have also been reported to exhibit particle sizes in the range of approximately 900–1000 nm. The comparatively larger size was attributed to particle aggregation influenced by green synthesis, in which bioactive compounds present in the extracts promote interparticle interactions, leading to cluster formation in colloidal systems [33,34]. Zeta potential analysis yielded a surface charge of −19 mV, indicating moderate colloidal stability. Although the negative surface charge provides partial/weak electrostatic stabilization, the magnitude is insufficient to prevent particle aggregation in suspension. DLS results represent nanoparticle aggregates rather than the size of the primary nanoparticle. Such aggregation may be due to the presence of a residual plant species, but this does not alter the nanoscale dimensions of the primary particles. To confirm the actual particle dimensions, HR-TEM analysis was performed, which revealed the nanometer-sized individual particles despite their tendency to aggregate in the aqueous media because HR-TEM directly measures the individual primary particles in the dry state. SEM results also revealed the nanosized individual particles. The observed aggregation in the suspension may influence the biological performance by reducing the accessible surface area and may affect cellular internalization. Those aggregated nanoparticles still exhibit significant biological activity because the aggregates consist of nanosized individual particles capable of entering the cells; furthermore, the release of Zn^2+^ ions contributes to the cytotoxicity. Although the aggregation represents a limitation in colloidal stability, it does not exclude the biological activity in the present study [35]. Tuning of nanoparticle size is required; further optimization in the synthesis process, such as plant extract concentration, reaction pH, temperature, reaction timing and drying, etc., will influence the particle size, nucleation, aggregation behaviour, and colloidal stability. Future studies will include optimization of these parameters and incorporating a proper stabilization agent to prevent aggregation in suspension.

Cytotoxicity assessment was performed for *Lantana camera* plant extract alone against the H460 cell line; there was no inhibition observed up to 200 µg/mL. H460 cell line was exposed to the standard drug doxorubicin as a positive control to validate the responsiveness of the cell line under the experimental condition. Normal lung epithelial cell line BEAS-2B was subjected to cytotoxic assessment with LCFAE, which showed 50% inhibition at 200 µg/mL, whereas in H460 cells, the cytotoxic assessment resulted in an IC_50_ value of 50 µg/mL. Dose-response curves were analyzed using nonlinear regression to determine the IC_50_ values. The calculated IC_50_ values at 95% confidence interval were 167.4 for BEAS-2B cells. In this comparison, the selectivity index (SI) was calculated as 3.35, indicating preferential cytotoxicity towards H460 cells compared with the normal cells BEAS-2B in the present experimental conditions. Although the SI value3.35 suggests favourable in vitro selectivity, around 50% inhibition was observed at the highest concentration (200 µg/mL). It should be noted that BEAS-2B cells are immortalized human bronchial epithelial cells and therefore may not fully recapitulate the biological characteristics of primary normal lung epithelial cells. Additional validation using primary normal lung cells and in vivo models will be necessary to further establish the therapeutic selectivity of the synthesized nanoparticles. Therefore, further validations are required regarding pharmacokinetic profiling, safe targeted delivery, long-term safety assessment, and systemic toxicity in the in vivo approach before considering their therapeutic applications. LC flower aqueous extract alone exhibited considerably lower cytotoxic potency, as evidenced by a much higher IC_50_ value of 446.91 µg/mL reported on the A549 lung cancer cell line by Raghu C et al., 2004 [36]. ZnO nanoparticles derived from *Solanum procumbens* demonstrated an IC_50_ of 61.28 µg/mL towards A549 cells [31]. Similarly, ZnO NPs derived from *Trichosanthes dioica* seed extract and *Euphorbia retusa* exhibited 51.4 µg/mL and 40 µg/mL against H460 cells, respectively [33,35]. The cytotoxic response observed in this current study is consistent with these reports. The results denoted that plant-mediated ZnO NPs exert enhanced anticancer activity at a lower concentration than compared to the plant extract. Viability checks and morphological investigations also corroborated the MTT-based cytotoxicity pattern. ZnO NPs treatment resulted in morphological alteration in a dose-dependent manner. The rounding and shrinkage of cellular morphology indicate the detachment of cells from the surface. Cell-to-cell interaction is mediated by integrins through linked kinases and focal adhesions. Apoptosis leads to a decrease in integrin function, resulting in the inactivation of linked kinases and focal adhesion [31,35,37]. The observed morphological changes are consistent with nanoparticle–cell interactions. Although the present study did not directly investigate the intracellular localization of LCFAE-ZnO NPs, previous studies have reported that ZnO NPs are commonly internalized by cells through endocytic pathways and may undergo partial dissolution within acidic intracellular compartments, thereby facilitating the release of Zn^2+^ ions and contributing to cell cytotoxicity. However, the visualization of direct internalization of ZnO NPs will be an important objective for future investigations [38,39].

Enzymes and oxidative stress markers are the main players in maintaining cellular homeostasis. Investigating these parameters is a critical indicator in the anticancer assessment. In the current study, nitric oxide (NO) release, lipid peroxidation (LPO), superoxide anion generation, and glutathione (GSH) levels were estimated in the untreated and treated groups. Dose-dependent increase in NO, LPO, and superoxide anion generation was observed, accompanied by a corresponding decrease in intracellular GSH, indicating oxidative stress. Conversely, *Solanum procumbens*-derived ZnO NPs suppress NO release in a dose-related manner in the A549 cell line, leading to slug inactivation and Bax activation specifically in A549 cells. The parameters NO, LPO, ROS and GSH are closely interconnected within cellular signalling pathways and modulation of ROS-GSH balance is commonly employed to evaluate the effectiveness of anticancer therapies [31]. Excessive superoxide anion generation is recognized as a primary trigger for cellular dysfunction, as it disrupts lipid metabolism and promotes peroxidative damage to membrane lipids, which leads to the loss of integrity of the mitochondrial membrane as well as the cellular membrane. Altered membrane integrity resulted in the loss of permeabilization equilibrium [31,37]. MMP fluorescence assay confirms mitochondrial membrane depolarization in a dose-related manner. The loss of MM integrity facilitates the cytochrome C release into the cytosol, which subsequently activates the caspase-dependent apoptotic cascade [37]. Similarly, Hoechst staining revealed that the DNA fragmentation upon treatment with LCFAE-ZnO NPs ultimately leads to cell death. Dutta et al. also reported that the *Trichosanthes dioica* seed extract-derived ZnO NPs exhibited dose-related DNA fragmentation by Hoechst staining on the H460 cell line [35,40]. The present study provided evidence supporting the anticancer activity of LCFAE-ZnO NPs against H460 cells. The NBT assay primarily measures the superoxide anions rather than the total ROS. Future studies employing DCFH-DA would provide a more comprehensive assessment of oxidative stress, and the observed regulation of apoptotic proteins is consistent with the activation of the mitochondrial-mediated apoptotic pathway, but the additional mechanistic studies involving caspase inhibition and cytochrome C release would further validate the proposed mechanism. Collectively, these findings support apoptotic cell death following treatment with LCFAE–ZnO NPs. However, the present study did not include quantitative apoptosis assays such as Annexin V-FITC or direct caspase activity measurements, which would provide more definitive quantification of apoptotic cell populations. Future studies incorporating these complementary assays will further validate and extend the mechanistic understanding of nanoparticle-induced apoptosis. The anti-proliferation property was confirmed by investigating cell proliferation using flow cytometry and cell cycle analysis. Results revealed that the DNA population gradually increased in the G0/G1 phase, demonstrating cell cycle arrest and supporting the anti-proliferative property of LCFAE–ZnO NPs. Deregulation of cell cycle control is a basic hallmark of cancer development. Cell cycle progression is tightly regulated by cyclin-dependent kinases (CDKs), whose activity depends on their interaction with specific regulatory cyclins and cyclin-dependent kinase inhibitors (CDKIs). The functions of CDKs are phase-specific, ensuring orderly cell cycle transition. Progression through the G1 phase is primarily governed by CDK4 and CDK6 in association with D-type cyclins, whereas the G1-S phase transition is regulated by CDK2 in complex with cyclin E. CDK2–cyclin A complexes facilitate DNA synthesis during the S phase, while the G2–M transition is controlled by CDK1 bound to cyclin B [37]. LCFAE–ZnO NPs exert growth-inhibitory effects by inducing cell cycle arrest at various checkpoints, predominantly in the G1, S, or G2/M phases.

As previously discussed, cytochrome c release activates the caspase cascade, leading to apoptosis, as confirmed by gene expression analysis. Apoptotic markers BAX and p53 showed upregulated expression, while the anti-apoptotic marker Bcl2 exhibited downregulated gene expression upon treatment with LCFAE-ZnO NPs. The altered Bax/Bcl-2 ratio favours mitochondrial outer membrane permeabilization, thereby amplifying cytochrome c release and reinforcing apoptotic signalling. Upregulation of p53 further supports this mechanism, as p53 plays a critical role in sensing oxidative stress and DNA damage, leading to transcriptional activation of pro-apoptotic genes [41]. These molecular events are consistent with the observed oxidative stress, mitochondrial dysfunction, and nuclear DNA fragmentation, collectively suggesting that LCFAE–ZnO NPs induce apoptosis responses associated with mitochondrial dysfunction.

Immunoblotting analysis of pro-apoptotic marker Bax and p53 was significantly upregulated with a marked reduction in anti-apoptotic protein Bcl-2. The tumor suppressor protein P53 plays an important role in regulating Bcl2 by maintaining mitochondrial membrane permeability, supporting cell viability, whereas Bax triggers apoptosis through disruption of mitochondrial membrane integrity and cytochrome c release [42,43,44,45]. A Western blot was performed for the selected concentrations to evaluate early molecular response; assessment of higher concentrations would provide additional dose-response regulation of apoptotic signaling. The inclusion of higher concentrations is an important direction for future investigations. Intracellular ROS generation increased on treatment with LCFAE-ZnO NPs due to oxidative stress-induced apoptosis. Previous studies by Akhtar et al., 2006 and Mohammadinejad et al., 2019 [46,47] reported the ROS-mediated intrinsic apoptotic pathway induction by green-synthesized ZnO Np treatment.

The present findings are consistent with previous reports demonstrating the anticancer potential of green-synthesized ZnO-based nanomaterials against lung cancer cells. Dutta et al. reported that biosynthesized ZnO–Ag nanoparticles exhibited significant cytotoxicity toward NCI-H460 cells by promoting oxidative stress and apoptosis while also demonstrating antimicrobial and photocatalytic activities. Similarly, the *Lantana camara* flower-mediated ZnO nanoparticles synthesized in the present study induced marked cytotoxicity, increased superoxide generation, altered apoptosis-related protein expression, and promoted apoptotic cell death in NCI-H460 cells. Although the study by Dutta et al. employed Ag-doped ZnO nanoparticles, whereas the present work utilized pure ZnO nanoparticles synthesized using *Lantana camara* flower extract, both studies support the therapeutic potential of green-synthesized ZnO-based nanomaterials against lung cancer cells. Furthermore, the present study extends these findings by providing a detailed mechanistic evaluation involving oxidative stress, apoptosis, cell cycle regulation, and apoptosis-associated protein expression [35]. However, the present study has certain limitations. Although LC-MS analysis identified bioactive constituents in the LC flower extract prior to nanoparticle synthesis, the surface chemistry of the final calcined ZnO Nps was not directly characterized. Since calcination was performed at 400 °C for 2 h, the persistence of plant-derived organic species on the nanoparticle surface cannot be confirmed from the present data, and therefore the observed biological effects are not attributed to retained phytochemical surface capping. Additional investigations involving detailed surface characterization using techniques such as XPS, TGA, or elemental analysis together with comprehensive in vivo studies are required.

Conclusively, the anticancer potential of LC flower aqueous extract-mediated zinc oxide nanoparticles (LCFAE–ZnO NPs) was convincingly explored through multiple validated biological parameters, demonstrating their ability to inhibit lung cancer cell growth. Although these findings suggest that the synthesized nanoparticles may be a candidate against lung cancer cells, further investigations are needed, including biodistribution studies, pharmacokinetics, long-term biocompatibility, evaluation of systemic toxicity, and validation in an appropriate in vivo lung cancer model to establish their safety and efficacy.

## 4. Materials and Methods

### 4.1. Lantana Camara Flower Aqueous Extract

*Lantana camara* flowers were obtained from the local area of Saudi Arabia. Flowers were rinsed in water and dried in the shade. 10 g of dried flower powder was mixed with 100 mL of distilled water and stirred continuously for 4 h. The solution was filtered using Whatman filter paper No.1. The filtrate was retained for the ZnO nanoparticle synthesis. LC aqueous plant extract was subjected to LC/MS to investigate the presence of phytocompounds. Data acquisition and processing were conducted using MassLynx v4.1 software (Waters Corporation, Milford, MA, USA). Databases used were massBank, MEDLIN, ChemSpider, and published literature. Mass accuracy tolerance was ±5 ppm.

### 4.2. LCFAE-Mediated ZnO Np Synthesis

10 mL of the flower extract was taken and mixed with 0.02 M zinc acetate (Sigma-Aldrich, St. Louis, MO, USA). pH of the mixture was maintained around 12 by adding Sodium hydroxide (Sigma-Aldrich, St. Louis, MO, USA). After that, the solution was stirred at 1000 rpm for 3 h, resulting in a gradual color change, and the white precipitate formed was centrifuged at 3500 rpm for 5–10 min. Then, an ethanol (Sigma-Aldrich, St. Louis, MO, USA) wash was given to remove any impurities, and the washed particles were calcined at 400 °C for 2 h. Finally, the calcined NPs were subjected to characterization.

### 4.3. Characterization

LCFAE-mediated ZnO NPs were characterized to understand and acknowledge their physicochemical properties. Firstly, basic confirmation by UV wavelength scanning was performed on the Cary 60 (version 2.00) (Agilent Technologies, Santa Clara, CA, USA) between 200 and 800 nm. For this, the sample was prepared at 1 mg/mL and sonicated. The homogeneous sample was then subjected to UV spectroscopic analysis to check the specific peak value range of synthesized LCFAE-ZnO NPs. FT-IR assessment was performed, followed by UV analysis to determine the presence of phytoactive functional groups merging between plain LCFAE and Vs. LCFAE-ZnO NPs. FT-IR analysis was carried out using IRspirit_DESKTOP-4UREJCM-instrument (Shimadzu Corporation, Kyoto, Japan) between 340 and 4700 nm range, Happ-Genzel used as apodization, and the resolution was 2 cm^−1^. Surface topography was analyzed by field-emission scanning electron microscopy—FEI QUANTA 250 FEG, Thermo Fisher (Hillsboro, OR, USA). Images of various magnifications have been captured, 20.00 KV energy has been used with high vacuum conditions, and also the elemental configuration was ruled out by EDS analysis. The crystallographic nature was investigated with the help of the Empyrean Series III Diffractometer (Malvern Panalytical, Malvern, UK) with 450 eV energy resolution. The source of X-rays was Cu Kα (λ = 1.54 Å). The size of LCFAE-mediated ZnO NPs was analyzed using an Anton Paar particle size analyzer (Litesizer 500) (Graz, Austria) at 25 °Cand 10 s difference in each run with 1.3303 refraction index. Zeta potential analysis was also assessed by an Anton Paar Zeta potential analyzer (Litesizer 500) at 25 °C. The crystallographic nature was investigated with the help of the Empyrean Series III Diffractometer with 450 eV energy resolution. From this physicochemical characterization, a deep understanding of the synthesized ZnO NPs was observed. High-resolution transmission electron microscopy (HR-TEM) (JEOL, Tokyo, Japan, JEM-2100 plus imaging) was performed to investigate the individual particle size and morphological status of LCFAE-ZnO NPs.

### 4.4. In Vitro Studies

#### 4.4.1. Cell Culture

H460 cell line—Human non-small cell lung cancer cell line and BEAS-2B cell line—Human lung epithelial cell line were purchased from ATCC (Manassas, VA, USA). STR authentication is available from ATCC in the USA. We tested for Mycoplasma using the PCR Mycoplasma Detection Set, (TaKaRa Bio Inc., Kusatsu, Shiga, Japan) and found it negative. The cells were maintained by 10% Dulbecco’s Modified Eagle’s medium under a 37 °C CO_2_ incubator (Thermo Fisher Scientific, Waltham, MA, USA) with a humidified chamber. Monolayered cells were subcultured for further processing.

#### 4.4.2. Anti-Proliferative Property Assessments

The stock of LCFAE-ZnO NPs was prepared in sterile water at 1 mg/mL for the following anti-proliferative assessments: cytotoxicity by MTT, viability confirmation by TBE (Trypan Blue Exclusion), and structural deformities analysis by Phase-contrast microscopy (Olympus Corporation, Tokyo, Japan) [48,49,50]. Initially, BEAS-2B and H460 cells were seeded in a 96-well cell culture plate as per standard protocol and incubated for maximum confluency. The media was then replaced with the sample-containing media (2%), which was serially diluted using the half-concentration method. The added concentration ranged from 0.2 to 800 µg/mL for BEAS-2B and 0.2–100 µg/mL for H640 cells, respectively. The cells were incubated for 24 h to assess the effect. After the experimental duration, the cells were washed with PBS to remove the residual drug and media compounds. Then, MTT (5 mg/mL of 1XPBS) containing media (2%) was added and kept for 2–4 h at 37 °C. The media with MTT was removed, and the stain was dissolved from the cell wall of viable cells with the help of a saturated DMSO solution of 200 µL. The bluish-purple solution was read using an ELISA reader (BioTek Instruments, Inc., Winooski, VT, USA) at 490 and 630 nm. The % inhibition was calculated by comparing treated cells with the untreated control, and the graph was plotted. Percentage inhibition was computed by the following formula:

% Inhibition = [1 − ((ABS_T490_ − ABS_T630_)/(ABS_C490_ − ABS_C630_)) × 100]

Cells in an appropriate number were seeded for the TBE assay. Cells were treated at 70–80% confluency with ½ IC_50_, IC_50_, and 2 IC_50_, such as 25, 50, and 100 µg/mL for 24 h. Then, the cells were collected by trypsinization, washed with PBS and stained with Trypan Blue. The viable and non-viable cells were counted. Viable cell percentage was calculated by dividing the viable cell count by the total cell count, multiplied by 100, as follows,

% Viability = [Total number of live cells/Total number of live and dead cells] × 100

Similarly, treated and untreated groups of cells were examined under phase-contrast microscopy for the morphological alterations upon treatment with LCFAE–ZnO NPs of different concentrations. The images were recorded for the cellular detachments, cell wall disintegration, vacuole formations, cellular shrinkages, nuclear damage, etc.

### 4.5. Antioxidant Enzymes

The morphological deterioration indicates the impairment of oxidative balance. Hence, the primary enzymes responsible for oxidative balance were studied, including Nitric oxide (NO), Lipid peroxidase (LPO), and Glutathione reductase (GSH), and the Reactive oxygen species (ROS) level was also assessed for a better understanding [51,52,53,54].

NO was estimated using the Griess method by Griess A (1% sulfanilamide) and Griess B (0.1% NED) reagents (Sigma-Aldrich, St. Louis, MO, USA). The supernatant of 100 µL was collected from the cells treated with LCFAE–ZnO NPs for 24 h and also from the untreated group. In total, 50 µL of Griess A was added and incubated for 5 min, and 50 µL of Griess B was added and incubated for 10–15 min of incubation in the dark. The solution mixture was then subjected to an absorbance reading at 540 nm. The values were compared with the standard curve and interpreted. Since NO was estimated from the supernatant, the ZnO NPs alone group was kept as a nanoparticle control to avoid nanoparticle interference.

The lipid peroxidation protocol was followed by Ohkawa et al., 1979 [52]. With brief modification as follows, treated and untreated cells were collected using trypsin, washed with PBS, then homogenized with cold KCl buffer (Sigma-Aldrich, St. Louis, MO, USA). To 100 µL of cell homogenate, 20% Acetic acid (Sigma-Aldrich, St. Louis, MO, USA) (pH 3.2) of 1.5 mL, TBARS (0.8%) (Sigma-Aldrich, St. Louis, MO, USA) of 1.5 mL, SDS (8.1%) (Sigma-Aldrich, St. Louis, MO, USA) of 200 µL, and remaining water (0.7 mL) were added. The mixture and blank were kept in a 100 °C water bath for 60 min, then the tubes were cooled down. The mixture was then subjected to absorbance reading against a blank at 532 nm. The optical density values obtained were plotted in the standard graph of MDA, and concentrations were computed.

Cells were seeded in 96-well cell culture plates and treated with LCFAE–ZnO NPs for 24 h at their maximum confluency. The treated and control cells were washed with 1× PBS twice to remove the drug and subjected to 0.1% NBT treatment for 60 min at room temperature in the dark. Then, the cells were washed with 70% methanol after the removal of the NBT solution. The stain was solubilized using 120 µL of 2 M KOH and 120 µL of DMSO (Sigma-Aldrich, St. Louis, MO, USA), which was also retained for the blank. The absorbance was read at 630 nm and then compared with the control group to obtain the percentage of ROS release as follows,

% ROS release = [Difference between OD of treated and untreated/OD of untreated] × 100

Cells were collected from treated and control cells by trypsinization, washed with PBS and cell homogenate was prepared using cold 1× PBS. 500 µL of homogenate was mixed with 500 µL of 4% sulfosalicylic acid (Sigma-Aldrich, St. Louis, MO, USA) and kept at 4 °C for 60 min. Then, the solution was centrifuged and 33 µL of supernatant was mixed with 66 µL of DTNB (Sigma-Aldrich, St. Louis, MO, USA) and 900 µL of 0.1 M Potassium Phosphate buffer. The solution was read at 412 nm. The values were calculated and expressed as µM sulfhydryl units.

### 4.6. Apoptosis

#### 4.6.1. Hoechst Staining

Treated and untreated groups of cells were subjected to Hoechst staining to investigate DNA stability. Initially, cells were washed with PBS to remove the residual drug and media components and fixed with 4% formaldehyde for 15–20 min. The cells were washed with PBS, and Hoechst stain (Sigma-Aldrich, St. Louis, MO, USA) of 5 µg/mL was used and incubated in the dark for 5–10 min. Then, the stained cells were washed once again with PBS to remove the excessive Hoechst stain. Fluorescence microscopy (Olympus Corporation, Tokyo, Japan) was used to observe the stained cells and photographed [55].

#### 4.6.2. MMP Staining

Mitochondrial membrane potential was determined by the Rhodamine 123 staining assay. Media was discarded from the treated and untreated cells. Cells were washed with PBS to thoroughly remove the medium. Then, 10 µM Rhodamine 123 (Cayman Chemical Company, Ann Arbor, MI, USA) was added to the cells and incubated for 30 min in the dark. The stained cells were washed to remove the extra stain. Then the stained cells were analyzed under fluorescence microscopy and photographed [56].

#### 4.6.3. Flow Cytometry

Cellular DNA content accumulation was investigated by cell cycle analysis using Flow cytometry. Cells were treated with LCFAE–ZnO NPs. Treated and untreated cells were collected by trypsinization, washed with PBS, and 70% cold ethanol was added to prepare a single-cell suspension and kept at 4 °C overnight. Then, the cells were treated with RNase buffer (0.1 mg/mL) and stained with 0.5 mg/mL of PI stain for half an hour. The individual nuclei of PI-stained cells were detected using a flow cytometer (BD FACS Calibur, Becton Dickinson, San Jose, CA, USA), and the data were analyzed using Cell Quest Pro V 3.2.1 software. For cell cycle analysis, approximately 10,000 events were acquired for each sample. Cell debris was excluded by appropriate forward- and side-scatter gating. Doublet discrimination was performed to exclude cell aggregates, and only singlet cells were included for subsequent analysis of the G0/G1, S, G2/M, and sub G1 cell populations [57].

#### 4.6.4. Gene Expression by qPCR

Molecular-level investigation was performed using real-time PCR for the apoptosis-related genes. Total RNA was isolated from treated and control cells with the help of RNA Isoplus reagent. cDNA synthesis was carried out using Prime Script 1st Strand cDNA Synthesis Kit, TaKaRa (Cat.No: 6110A) (San Jose, CA, USA) as per the manufacturer’s protocol. Briefly, RNA, dNTP, oligo dT primer, and RNase-free water were mixed together as mentioned in the protocol and kept at 65 °C for 5 min. Then, after cooling, the mixture was added with the next set of reagents, like the primer mixture, reverse transcriptase, RNase inhibitor, buffer, as instructed by the protocol of the kit, and incubated at 43 °C for 45 min. The synthesized cDNA was subjected to gene expression analysis using a Bio Rad real-time PCR system by employing the TB green @ premix kit, TaKaRa (Cat. No: RR820A) (Kusatsu, Japan). The contents were mixed as per the instructions provided in the protocol manual. cDNA, forward and reverse primers, TB Green Premix Ex Taq II, ROX Reference Dye, and sterilized distilled water were added according to the manufacturer’s protocol. Gene expression of apoptotic and anti-apoptotic markers such as p53, BAX, and Bcl2, respectively, was evaluated with the housekeeping gene Beta-actin. Annealing temperature and oligo sequences are provided in Table 3. The conditions of the PCR cycle consist of 3 min—denaturation at 95 °C, 30 s—annealing at 60 °C, and 30 s—extension at 72 °C. Gene expression was evaluated by the 2^−∆∆Ct^ method.

#### 4.6.5. Western Blot Assay

The treated and untreated H460 cells were collected with the help of a cell scraper and washed thoroughly with Tris-buffered saline (TBS). The cell suspension was collected in precooled Eppendorf tubes and centrifuged at 1000 rpm. The pellet obtained was washed with PBS and added to the TOTEX lysis buffer (20 mM HEPES, 350 mM NaCl, 20% glycerol, 1% Nonidet P40, 1 mM MgCl_2_, 0.5 mM EDTA, 0.5 mM DTT, and 0.1% PMSF) at a ratio of 1 mL per 4 × 10^6^ cells. The lysates obtained were centrifuged at 16,000× *g* for 20 min at 4 °C. The supernatant (which contains the protein) was subjected to a protein estimation assay. Approximately 20 µg of protein from the samples was added with an equal volume of 2× Laemmli sample buffer, heated at 95 °C, centrifuged at 16,000× *g* and then loaded for electrophoresis using SureCast™ acrylamide reagent (10% resolving gel and 5% stacking gel). B-actin was used as a loading control (n = 3, biological replicates). Then, the proteins were transferred to a nitrocellulose membrane using a Mini Blot Module (Invitrogen™ B1000, Thermo Fisher Scientific, Waltham, MA, USA) at 10 V for one hour. The membrane was then washed with TBST and blocked with 5% bovine serum albumin prepared in TBST for one hour at room temperature. Later, the primary antibody diluted to 1:1000 was added to the membrane and incubated overnight at 4 °C. The next day, the membrane was washed 3 times with TBST and incubated with HRP-conjugated secondary antibodies (Abcam Ltd, Cambridge, UK) at 1:2000 for 1 h. Later, the membrane was washed with TBST 5 times, and the protein was visualized with Promega enhanced chemiluminescence (ECL) Western blotting substrate. The Bio-Rad Laboratories ChemiDoc imaging system.

(Bio-Rad Laboratories, Inc., Hercules, CA, USA) was used for visualization and capturing the image. The normalization procedure was carried out by dividing the target protein intensity by the loading control intensity [58].

### 4.7. Statistical Investigation

Experiments were triplicated, and data were provided as Mean ± SD. GraphPad Prism software 5.1 was utilized to analyze the statistical relationship between the untreated and treated groups. One-way ANOVA was performed, and the groups were compared using Tukey’s multiple comparison test. *p* < 0.05 is considered statistically significant.

## 5. Conclusions

In the present study, ZnO NPs were successfully synthesized via a green, biogenic approach using *Lantana camara* flower aqueous extract. Comprehensive physicochemical characterization confirmed the formation of nanocrystalline ZnO, indicating the role of floral biomolecules in nanoparticle stabilization. The biosynthesized ZnO nanoparticles exhibited pronounced cytotoxic activity against human non-small cell lung cancer (NCI-H460) cells in a dose-dependent manner. Mechanistically, ZnO nanoparticle exposure induced significant oxidative stress, accompanied by mitochondrial membrane depolarization and nuclear DNA damage, collectively suggesting the activation of apoptotic pathways. Flow cytometric analysis further disclosed G0/G1 phase cell cycle arrest, indicating inhibition of cancer cell proliferation. At the molecular level, the upregulation of Bax, the p53 gene, and concurrent downregulation of the Bcl-2 gene suggest the involvement of mitochondrial dysfunction. Protein-level investigation through Western blotting further confirms the apoptotic pathway. Overall, these findings provide preliminary evidence supporting the potential of biosynthesized ZnO NPs as candidates for further investigation in lung cancer research. Nevertheless, further in vivo validation and detailed mechanistic investigations are warranted to establish their safety, pharmacokinetics, and translational therapeutic applicability.

## Figures and Tables

**Table 1:** Positive Ionization mode of phytoactive compounds.

Compound	*m*/*z*	RT (min)	Adduct	Tentative Structure Class
12-Oxo-phytodienoic acid	265.18	17.88	[M+H]^+^	Oxylipin (fatty acid derivative)
13-epi-12-oxo phytodienoic acid	293.21	16.57	[M+H]^+^	Oxylipin
13Z-Docosenamide	338.35	25.85	[M+H]^+^	Fatty acid amide
Podocarpane derivative	243.14	21.12	[M+H]^+^	Diterpenoid
2-Acetyl tetrahydroxybutyl imidazole	231.08	1.08	[M+H]^+^	Nitrogen heterocycle
3-Phenylpropanal	135.08	21.14	[M+H]^+^	Aromatic aldehyde
4-oxo-2-Nonenal	155.10	14.76	[M+H]^+^	Lipid peroxidation product
4-Oxoisotretinoin	315.20	19.34	[M+H]^+^	Retinoid
6-Gingerol	295.19	17.69	[M+H]^+^	Phenolic ketone
α-Asarone	209.11	15.74	[M+H]^+^	Phenylpropanoid
Benzaldehyde	107.05	15.33	[M+H]^+^	Aromatic aldehyde
Caprylic acid	145.12	16.23	[M+H]^+^	Medium-chain fatty acid
Confertifoline	235.17	19.83	[M+H]^+^	Sesquiterpene
Dehydroabietic acid	301.22	21.88	[M+H]^+^	Diterpenoid
Dehydrovomifoliol	223.13	15.13	[M+H]^+^	Apocarotenoid
Dihydroactinidiolide	181.12	14.59	[M+H]^+^	Norisoprenoid
Dimethyl fumarate	145.05	1.32	[M+H]^+^	Ester
Docosahexaenoyl glycine	386.26	16.06	[M+H]^+^	Fatty acid conjugate
Fenuron	165.09	13.47	[M+H]^+^	Urea derivative
Isopeonol	167.07	17.97	[M+H]^+^	Phenolic compound
Kynuramine	165.09	19.43	[M+H]^+^	Tryptophan metabolite
Methyl jasmonate	225.15	15–18	[M+H]^+^	Jasmonate (plant hormone)
Methylisoeugenol	179.11	16.55	[M+H]^+^	Phenylpropanoid
Nonanoic acid	159.14	17.79	[M+H]^+^	Fatty acid
Oleamide	282.28	25.17	[M+H]^+^	Fatty acid amide
Palmitic amide	256.26	25.23	[M+H]^+^	Fatty acid amide
Solasodine	414.33	21.95	[M+H]^+^	Steroidal alkaloid
trans,trans-Farnesol	223.21	22.58	[M+H]^+^	Sesquiterpenoid alcohol
Tricosanoic acid methyl ester	369.36	25–27	[M+H]^+^	Long-chain fatty ester
Tryptamine	161.10	11–20	[M+H]^+^	Indole alkaloid

**Table 2:** Negative Ionization mode of phytoactive compounds.

Compound	*m*/*z*	RT (min)	Adduct	Putative Structure Class
(S)-2-Hydroxyglutarate	147.03	1.1	[M−H]^−^	Dicarboxylic acid
3-Hydroxy-3-methylglutaric acid	161.05	1.05	[M−H]^−^	Organic acid
2,5-Dihydroxybenzaldehyde	137.02	11.03	[M−H]^−^	Phenolic aldehyde
9-cis-Retinoic acid	299.19	21.98	[M−H]^−^	Retinoid (terpenoid)
Iso-Olomoucine	297.14	18.64	[M−H]^−^	Alkaloid/CDK inhibitor
Pentalenic acid	249.14	21.68	[M−H]^−^	Sesquiterpenoid acid

**Table 3:** Primer sequence.

Sl No	Gene	Primer Sequences F: 5′-3′ and R: 5′-3′	Temperature
1	*Beta-actin*	F: GCAGATGTGGATCAGCAAGC R: GCAGCTCAGTAACAGTCCGC	*54.2*
2	*Bcl-2*	F: TACCTGAACCGGCACCTG R: GCCGTACAGTTCCACAAAGG	*54.2*
3	*p53*	F: CTCTGACTGTACCACCATGG R: CCGTCCCAGTAGATTACCAC	*54.2*
4	*BAX*	F: CCTGCTTCTTTCTTCATCGG R: AGGTGCCTGGACTCTTGGGT	*54.2*

## Data Availability

All data analyzed and used during the current study will be available from the corresponding authors upon reasonable request.

## Abbreviations

The following abbreviations are used in this manuscript: ZnO NPs—Zinc oxide nanoparticles; FTIR—Fourier transform infrared spectroscopy; SEM—Scanning electron microscopy; XRD—X-ray diffraction; PSA—Particle size analysis; qRT-PCR—Quantitative real-time PCR; WHO—World Health Organization; TKIs—Tyrosine kinase inhibitors; LC—*Lantana camara* L.; NSCLC—Human non-small cell lung cancer; NO—Nitric oxide; LPO—Lipid peroxidation; ROS—Reactive oxygen species; GSH—Reduced glutathione; MMP—Mitochondrial membrane potential.
